# Zinc-doped Prussian blue enhances photothermal clearance of *Staphylococcus aureus* and promotes tissue repair in infected wounds

**DOI:** 10.1038/s41467-019-12429-6

**Published:** 2019-10-03

**Authors:** Jun Li, Xiangmei Liu, Lei Tan, Zhenduo Cui, Xianjin Yang, Yanqin Liang, Zhaoyang Li, Shengli Zhu, Yufeng Zheng, Kelvin Wai Kwok Yeung, Xianbao Wang, Shuilin Wu

**Affiliations:** 1 School of Materials Science and Engineering, The Key Laboratory of Advanced Ceramics and Machining Technology by the Ministry of Education of China, Tianjin University, 300072 Tianjin, China; 2 Ministry-of-Education Key Laboratory for the Green Preparation and Application of Functional Materials, Hubei Key Laboratory of Polymer Materials, School of Materials Science and Engineering, Hubei University, 430062 Wuhan, China; 3 State Key Laboratory for Turbulence and Complex System and Department of Materials Science and Engineering, College of Engineering, Peking University, 100871 Beijing, China; 4 Department of Orthopaedics and Traumatology, Li Ka Shing Faculty of Medicine, The University of Hong Kong, Pokfulam, Hong Kong, 999077 China

**Keywords:** Biomaterials, Bacterial infection, Biomedical engineering, Metal-organic frameworks

## Abstract

The application of photothermal therapy to treat bacterial infections remains a challenge, as the high temperatures required for bacterial elimination can damage healthy tissues. Here, we develop an exogenous antibacterial agent consisting of zinc-doped Prussian blue (ZnPB) that kills methicillin-resistant *Staphylococcus aureus* in vitro and in a rat model of cutaneous wound infection. Local heat triggered by the photothermal effect accelerates the release and penetration of ions into the bacteria, resulting in alteration of intracellular metabolic pathways and bacterial killing without systemic toxicity. ZnPB treatment leads to the upregulation of genes involved in tissue remodeling, promotes collagen deposition and enhances wound repair. The efficient photothermal conversion of ZnPB allows the use of relatively few doses and low laser flux, making the platform a potential alternative to current antibiotic therapies against bacterial wound infections.

## Abstract (web summary)

Here, the authors apply transition metal doping in combination with phototermal therapy to treat *Staphylococcus aureus*-infected wounds, and show that release of ions by local heat enhances bacteria clearance and promotes tissue repair in a rat model of MRSA-infected wounds

## Introduction

Bacterial infectious disease presents an increasing threat to global healthcare and is the leading cause of mortality and morbidity worldwide, claiming ~15 million lives each year^1–5^. Moreover, the rapid emergence of drug-resistant bacterial infections has resulted in an enormous public medical and financial burden since the long-term abuse of antibiotics is driving the rapid evolution, rise and spread of antibiotic-resistant pathogenic bacteria at alarming rates^1,2,5^. In particular, the daunting challenge in the treatment of *Staphylococcus aureus* (*S. aureus*) is the rising prevalence of methicillin-resistant *S. aureus* (MRSA) strains in chronic or recurrent infections, which exhibit high levels of tolerance to clinically available antibiotics^4^. The number of deaths associated with MRSA exceeds those caused by hepatitis, HIV/AIDS and influenza combined^1^. Moreover, many conventional antibacterial methods, including silver nanoparticles, are becoming less efficient because bacteria can develop resistance to silver nanoparticles^6^. Consequently, the development of excellent antibacterial therapeutics is urgently necessary to effectively and safely fight multi-drug-resistant bacterial infections, including MRSA infections.

More recently, photothermal therapy (PTT) based on near-infrared (NIR) light irradiation has been widely applied^6–8^. The locally increased temperature (more than 50 °C) generated by NIR light irradiation can lead to bacterial death through hyperthermia-induced denaturation of bacterial proteins and irreversible bacterial destruction^9^. Local heat triggered by photothermal effects will damage the bacterial membrane and cause an increase in its permeability^9,10^. Simultaneously, NIR light for PTT exhibits an excellent capability to penetrate tissue with minimal damage to healthy tissues. Compared with current antibiotic therapies, PTT has broad-spectrum bactericidal efficiency through physical heat, and relatively brief treatment (only a few minutes) can efficiently kill bacteria^10,11^. Consequently, PTT for bacterial infections by NIR light irradiation has tremendous advantages for combating drug-resistant bacterial infections, including rapid MRSA eradication, even for MRSA biofilm eradication. However, when the increased temperature caused by PTT is too high for complete bacterial elimination, non-localized heat and hyperthermia usually result in great damage to healthy tissues^11^. On the other hand, it is difficult to achieve significant photothermal ablation of bacteria at relatively low temperatures. Therefore, safe (minimal invasion to healthy tissues) and effective bacterial destruction by PTT alone remains challenging. The key point is to allow the synergistic antibacterial action through PTT and other methods to enhance the photothermal ablation of bacteria.

Metal-organic frameworks (MOFs) represent a stable class of crystalline porous coordination compounds and can be assembled in a highly modular manner from metal ions and polydentate organic ligands; these materials possess controllable and ordered arrangements of multiple photonic unitsallowing the realization of good photonic functional applications^12–14^. The deliberate design and construction of photonic MOFs to improve and optimize the photonic functionality of photothermal theranostics is a promising prospect. That is, the structural tunability of photonic MOFs by various doping methods can be used to manipulate their light-harvesting ability, which is beneficial for fully understanding the mechanism of energy transfer processes within a framework. Prussian blue (PB) and its analogues are an important category of MOFs.

PB, which has been approved by the US Food and Drug Administration, has been applied in clinical treatment due to its excellent biocompatibility and biosafety^15,16^. PB has been used for PTT to generate heat under NIR light irradiation to induce localized damage to tumours, with minimal effect on normal biological tissue^15,16^. However, the unsatisfactory photothermal therapeutic efficiency of PB originates from the relatively poor photothermal conversion efficiency because the maximum NIR absorption peak of PB cannot be optimized to be near the wavelength of the NIR laser^15^. Consequently, the optimized design of PB derivatives with tailored optical properties through the variation in ion dopant density with various doping levels is highly promising. The tuneable bandgap transition of PB derivatives is expected to gradually redshift and move close to the near-IR regime. Additionally, the ionic dopant hybridization with bands of the host lattice of PB, as well as the interplay of crystal structure, determines the localized surface plasmon resonance (LSPR) characteristics, and a proportional change in carrier concentration will cause a significant shift in the LSPR optical behaviour and affect its photothermal conversion efficiency. Zinc is an essential trace element in the human body and a certain concentration of zinc ions exhibits good bioactivity and is central to many important physiological processes. In addition, both Zn and Fe are transition metals and have close atomic numbers, and Zn has a higher number of valence electrons than Fe does. Therefore, the doping of Zn in PB to replace Fe^2+^ has great potential to regulate the optical behaviour and bioactivity of PB.

In this work, by tuning the space unit of PB with various Zn doping levels with the guidance of density functional theory (DFT) calculations, the synergistic antibacterial effects of both photothermy and released ions of ZnPB are optimized for the therapy of MRSA-infected wounds. Specifically, the geometrical and electronic structure modelling of ZnPB with various doping levels by theoretical calculations is used to guide experimental design to optimize the Zn doping density for enhanced photothermal properties. The mechanism of the enhanced photothermal conversion efficiency of ZnPB is ascribed to the bandgap-narrowing effect and the redshifted LSPR towards lower energies with increasing Zn doping density. At the same time, the relative long-term biodegradability of ZnPB is illustrated by the characteristics of short-term rapid release from the interstitial sites and long-term stable release from the lattice sites. The mechanism underlying the broad-spectrum bactericidal action of ZnPB-3 (Zn-doped PB with the highest density in this work) originates from synergistic effect heat and ion release. That is, the photothermal effect of ZnPB-3 will effectively damage the bacterial membrane and cause its permeability to increase. Meanwhile, the local heat triggered by the photothermal effect accelerates the release of ions. The released Zn^2+^, Fe^2+^ and Fe^3+^ can rapidly penetrate bacteria, resulting in disturbance of the intracellular metabolic pathways of the bacteria and enhancement of the bactericidal efficiency. Additionally, the upregulated gene expression (*MMP-2*, *COL-I* and *COL-III*) and downregulated gene expression (*IL-1β*) caused by ZnPB-3 can increase the chemosynthesis of matrix metalloproteinases (MMPs), promote collagen deposition, and inhibit inflammatory factors to favour wound repair. The in vivo results with ZnPB-3 confirm its ability to relatively reduce MRSA infection and its effective antibacterial ability, and the released Zn^2+^ from ZnPB-3 can improve collagen deposition to favour wound healing. The detailed process is illustrated in Supplementary Fig. 1. This modified MOF system can be used as a valuable alternative to current antibiotic therapies against bacterial infection in the post-antibiotic era. Furthermore, this work provides a potential route for the optimized design of functional materials under the guidance of DFT.

## Results

### Theoretical calculations

Computational models based on DFT calculations using the Vienna Ab initio Simulation Package (VASP) can shed more light on the geometric and electronic structure modelling of ZnPB with various doping levels to guide the optimized design of PB derivatives with tailored optical properties. In the PB structure, metal centres of the same kind (Fe) with different electronic structures are classified as a class II mixed-valence compound^17^. The N-coordinated Fe has a charge of +3 (marked as Fe(III)-N, Fe(III) in red colour) and the C-coordinated Fe has a charge of +2 (marked as Fe(II)-C, Fe(II) in yellow colour). N-coordinated Fe(III) centres lie in the corners of a face-centred cubic (fcc) lattice of the crystal group F4̅3m^17^. When Zn(II) is doped into the lattice sites of PB, Zn(II) (marked in blue) will replace Fe(II) by cation exchange.

**Fig. 1:**
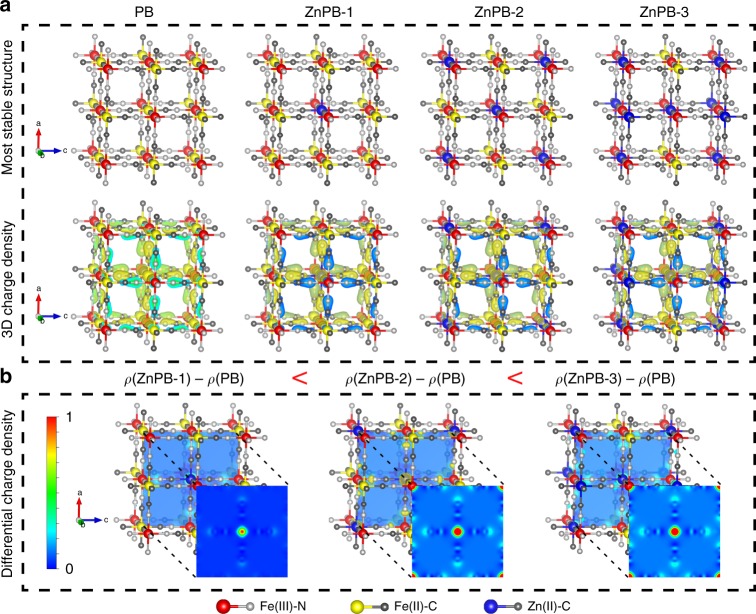
Theoretical calculations. **a** Optimized geometrical structure of PB and ZnPB with various doping levels (colour coding: Fe(III)-red, Fe(II)-yellow, Zn-blue, N-grayish and C-dark grey); 3D charge density difference plot of the optimization of PB and ZnPB**. b** 2D heatmap of differential charge density of PB and ZnPB in the midpoint of each unit cell

To theoretically simulate the optimized geometric structure of ZnPB with various doping levels, the relatively stable geometric structures are determined, as shown in Supplementary Figs. 2, 3. The corresponding total energy of each unit cell is calculated and compared. The lowest-energy structures with various doping levels are selected as the most stable geometric structures for further study, as shown in Fig. 1a and Supplementary Table 1. The three-dimensional (3D) charge density difference plot of the optimization of PB and ZnPB is calculated to further explore the electronic distribution and structure of ZnPB, and the charge distributions of PB and the different ZnPB samples exhibit similar shapes. The electron cloud of PB and different ZnPB is distributed primarily around the C and N atoms. Further details are listed in Supplementary Data 1. Figure 1b systematically reveals a regular change in the differential charge density as the dopant density changes, which is beneficial for guiding the establishment of structure–activity relationships. In particular, the two-dimensional (2D) heatmap of the differential charge density in the midpoint of each unit cell demonstrates that the differential charge density increases with increasing of Zn doping density. Therefore, the geometrical and electronic structure modelling of ZnPB with various doping levels provides the fundamental aspects of band structure modulation and LSPR to guide the optimized experimental design of PB derivatives with excellent photothermal properties.

### Morphology and structure

**Fig. 2:**
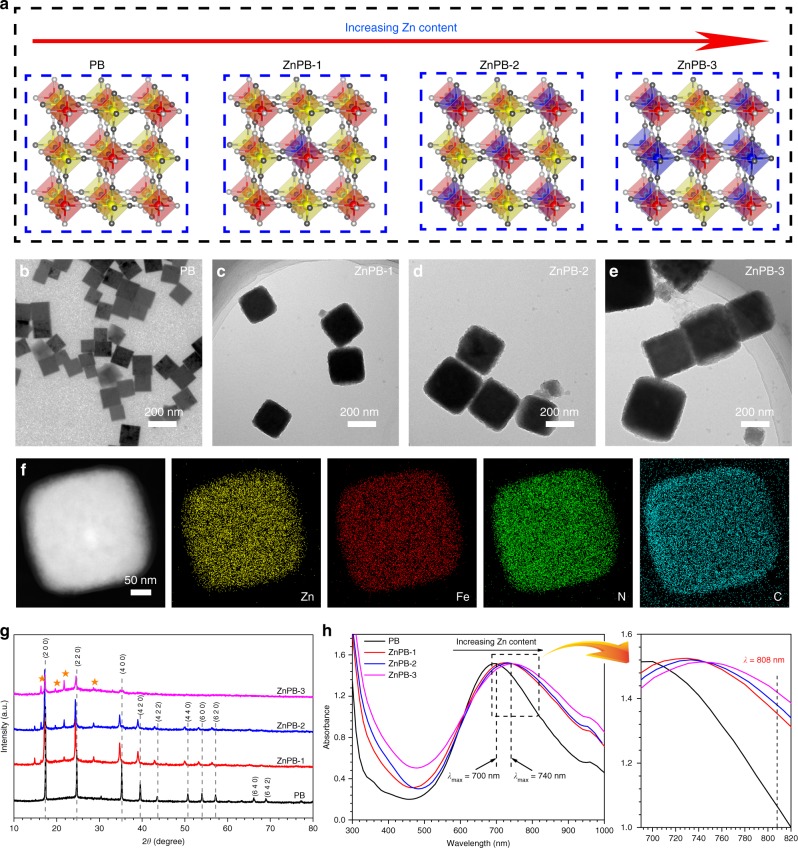
Morphology and structure. **a** Simplified geometrical structure of PB and ZnPB with various doping levels. TEM images of **b** PB, **c** ZnPB-1, **d** ZnPB-2 and **e** ZnPB-3. **f** Elemental mapping images of ZnPB-3. **g** XRD patterns of PB, ZnPB-1, ZnPB-2 and ZnPB-3 (Stellate signs present the peaks of zinc hexacyanoferrate). **h** UV–Vis–NIR spectra of PB, ZnPB-1, ZnPB-2 and ZnPB-3. Source data are provided as a Source Data file

According to the computational models in Fig. 1, the PB structure can be simplified into two octahedrally coordinated Fe centres (Fe(III)-N and Fe(II)-C) linked by cyanide bridges, whereas the ZnPB structure has an additional octahedrally coordinated Zn centre (Zn(II)-C), as shown in Fig. 2a. The fully optimized lattice constants (for PB, *a* = *b* = *c* = 10.05960 Å; for ZnPB-1, *a* = *b* = *c* = 9.998698 Å, for ZnPB-2, *a* = *b* = *c* = 10.132002 Å; and for ZnPB-3, *a* = *b* = *c* = 10.277851 Å) shown in Supplementary Table 1 vary with increasing of Zn doping density. With the guidance of the theoretical models, three different Zn doping density gradients are experimentally prepared in the process of forming PB. As shown in Fig. 2b through 2e, both PB and ZnPB exhibit a cubic structure with a corresponding average size varying from 100 to 400 nm. The possible mechanism explaining the the larger size of ZnPB compared to that of PB is that the substitution by doping of Zn ions distorts the host lattice and changes the interatomic coupling effects, which further impacts crystallographic phase stability during the growth of PB^18^. The elemental mapping images (Fig. 2f) of ZnPB-3 clearly exhibit the uniform distribution of individual elements of Zn, Fe, N and C, preliminarily confirming the successful doping of Zn into PB.

As shown in Fig. 2g, the X-ray diffraction (XRD) patterns of PB and ZnPB show characteristic peaks with high crystallinity corresponding to the (2 0 0), (2 2 0), (4 0 0), (4 2 0), (4 2 2), (4 4 0), (6 0 0), (6 2 0), (6 4 0) and (6 4 2) diffraction planes at 17.4°, 24.8°, 35.2°, 39.4°, 43.5°, 50.7°, 53.9°, 57.1°, 66.2° and 68.9°, respectively^15,16,19^. Compared with those of PB, the new diffraction peaks (marked by stellate signs) at 16.3°, 19.7°, 21.7° and 28.6° are indexed to zinc hexacyanoferrate^20^, indicating that Zn(II) is chemically bonded with [Fe(CN)_6_] to form -Fe-C≡N-Zn- in the reaction process and that Zn(II) is covalently bound to the cyanide ligands in the lattice sites. In addition, as the molar ratio of Zn/Fe increases, the characteristic peaks of PB in ZnPB gradually weaken or disappear, further suggesting that Zn(II) takes the place of Fe(II) to form zinc hexacyanoferrate. Moreover, the positions of the characteristic peaks of PB in ZnPB shift slightly towards lower angles, the result of which is ascribed to the larger diameter of high-spin Fe^2+^ compared to that of Zn^2+ ^^21^. Simultaneously, when the Zn/Fe molar ratio is increased gradually, the maximum absorbance of ZnPB NCs exhibits a redshift from 700 to 740 nm (Fig. 2h), and the maximum absorbances of PB, ZnPB-1, and ZnPB-3 are 700, 720, and 740 nm, respectively. The absorption spectra (300–1000 nm) of different concentrations of PB, ZnPB-1, and ZnPB-3 are shown in Supplementary Fig. 4. The redshift phenomenon can lead to the enhancement of NIR absorbance at 808 nm with increasing Zn content, which may improve the photothermal conversion under NIR light irradiation at 808 nm^15,22^. The related details and mechanisms will be discussed later.

### Photothermal properties

As demonstrated in Supplementary Fig. 5A–C, the temperature of the PB, ZnPB-1 and ZnPB-3 solutions in phosphate-buffered solution (PBS) gradually increases in a concentration-dependent and time-dependent manner under 808 nm NIR light irradiation (1.2 W cm^−2^). Specifically, according to Supplementary Fig. 5D, the maximum solution temperatures of 200 p.p.m. PB, ZnPB-1 and ZnPB-3 after 5 min increase to 60.16 ± 0.46 °C (*n* = 5), 61.4 ± 0.56 °C (*n* = 5, *P* < 0.01, compared with PB) and 63.4 ± 0.31 °C (*n* = 5, *P* < 0.001, compared with PB), respectively. In contrast, the temperature of pure PBS shows no significant change, suggesting that the presence of PB, ZnPB-1 and ZnPB-3 enables the rapid and efficient conversion of NIR energy into thermal energy. Moreover, excellent photothermal reversibility and cycling stability can be observed in Supplementary Fig. 5D. The recycling temperature variations of 200 p.p.m. PB, ZnPB-1 and ZnPB-3 were recorded under 808 nm NIR laser radiation for 5 min (laser on), followed by natural cooling to room temperature for 10 min (laser off) for five laser on/off cycles. The photothermal performance of PB, ZnPB-1 and ZnPB-3 shows no significant deterioration during five laser on/off cycles, highlighting the high stability and potential of PB, ZnPB-1 and ZnPB-3 as durable photothermal treatments. Meanwhile, ZnPB-3 solutions exhibit higher temperature increases than those of PB and ZnPB-1 solutions at the same mass concentration, clearly demonstrating that Zn-doped PB enhances the photothermal conversion efficiency. The performance of PB, ZnPB-1 and ZnPB-3 in converting the light into heat is quantitatively compared by photothermal conversion efficiency (*η*), which is calculated by the results of the time constant (*τ*_s_) and the maximum steady-state temperature (Supplementary Fig. 5E–G)^23,24^. The photothermal conversion efficiency (*η*) of ZnPB-3 (39.79%) is significantly higher than that of PB (22.91%) and ZnPB-1 (28.79%), further indicating that the photothermal conversion efficiency increases with increasing Zn doping density; the related mechanism will be discussed later.

### Mechanism of enhanced photothermal conversion efficiency

**Fig. 3:**
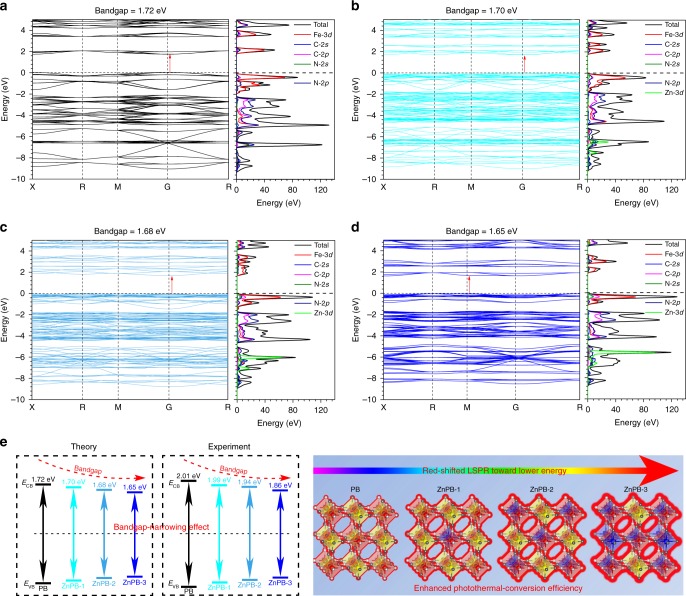
Mechanism of enhanced photothermal conversion efficiency. Theoretical calculations of band structure and density of states (DOS) of **a** PB, **b** ZnPB-1, **c** ZnPB-2 and **d** ZnPB-3. **e** Scheme of bandgap-narrowing effect with the increase of Zn-doped density by theory and experiment, and mechanism of enhanced photothermal conversion efficiency with the increase of Zn-doped density. Source data are provided as a Source Data file

The band structure of the semiconductor is central to the efficiency and mechanism of optical properties. PB has a strong charge-transfer absorption as a direct bandgap semiconductor at ∼1.75 eV^17^. To explore the electronic coupling and orbital contributions, the band structure and density of states of PB and ZnPB are calculated, as shown in Fig. 3a–d. The highest occupied orbital is selected as the Fermi energy level and is set to 0 eV as a reference. Obviously, the bandgap decreases from 1.72 to 1.65 eV with increasing Zn doping density. In addition, the valence band maximum (VBM) and conduction band minimum (CBM) are located at the G point for PB and ZnPB in the Brillouin zone, and the VBM and CBM of the PB and ZnPB are composed predominantly of Fe 3*d* states. Simultaneously, the VBM of ZnPB is occupied by Fe 3*d* states that are slightly hybridized with Zn 3*d* states, demonstrating that band edge modulation can be achieved in Zn-doped PB. According to the plot of the Kubelka–Munk function versus the bandgap energy from the ultraviolet–visible (UV–Vis) diffuse reflectance spectra in Supplementary Fig. 6, PB, ZnPB-1, ZnPB-2 and ZnPB-3 have bandgaps of 2.01, 1.99, 1.94, and 1.86 eV, respectively, which shows the same decreasing tendency as the theoretical calculations. Therefore, the bandgap-narrowing effect with increasing Zn doping density is verified both theoretically and experimentally (Fig. 3e). That is, introducing a higher concentration of Zn dopants in the PB lattice can give rise to a downward shift of the conduction band edge (*E*_CB_) and an upward shift of the valence band edge (*E*_VB_), leading to a net electronic bandgap-narrowing effect. The tuneable bandgap transition of ZnPB gradually redshifts and moves close to the NIR regime with increasing Zn doping density due to the lower bandgap energy. Accordingly, ZnPB possesses stronger NIR light absorption under 808 nm NIR light irradiation than PB does, thereby generating more heat energy (Supplementary Fig. 5).

According to the computational modelling of the electron distribution shown in Fig. 1, the electronic density increases with increasing Zn doping density, which leads to a change in the major electronic transition in the NIR regime from the bandgap to the LSPRs of ZnPB^25,26^. LSPR originates from the collective and coherent oscillations of the free carriers (namely, electrons or holes) in resonance with the incident light frequency^15,18^. The band structure modification of ZnPB will affect LSPR modulation^25,26^. Zn-doped PB exhibits tuneable LSPR characteristics through carrier density modulation, dependent on the size, dopant concentration, and dopant distribution inside the MOFs^25–27^. That is, Zn dopant hybridization with the bands of the host lattice of PB, as well as the interplay of the crystal structure, determines the LSPR characteristics, and a proportional change in the carrier concentration will cause a significant shift in the LSPR optical behaviour. The effect of LSPRs contributes to the NIR absorbance of both PB and ZnPB, which is mainly ascribed to the collective oscillations of free charge carriers of [Fe(CN)_6_] vacancies^15,18,27,28^. When more Zn ions occupy the lattice sites to form Fe−C≡N−Zn, the concentration of the [Fe(CN)_6_] vacancy will decrease, leading to the redshift of the LSPR peak towards lower energies. Additionally, the electronic transition between {Fe^III^[(t_2g_)3(e_g_)^2^]Fe^II^[(t_2g_)^6^]} and {Fe^II^[(t_2g_)^4^(e_g_)^2^]Fe^III^[(t_2g_)^5^]} results in NIR absorbance in PB^15,18,27,28^. Therefore, the presence of Zn ions in the lattice sites of PB will change the orbital energies and electron density of the cyanide bonds, which also leads to a redshift of the NIR absorbance peak of PB^15^. This is the reason why an increasing number of Zn ions present in the lattice sites of PB can optimize the NIR absorbance and enhance the photothermal conversion efficiency. In summary, the mechanism of the enhanced photothermal conversion efficiency of ZnPB is ascribed to the bandgap-narrowing effect and the redshifted LSPR towards lower energies with increasing Zn doping density. More importantly, the underlying mechanism of band structure modulation and LSPR can offer valuable insights for tailoring optical properties through theoretical and experimental understanding of the modulation principles.

### Ion release behaviour

**Fig. 4:**
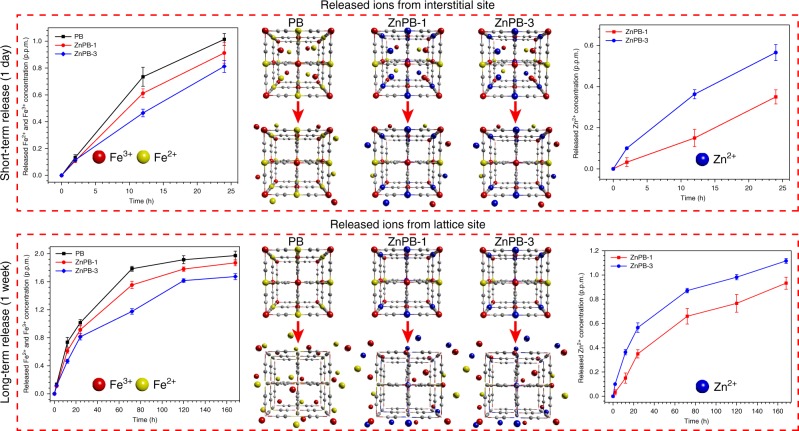
Ions release behaviour. Cumulative Fe^2+^, Fe^3+^ and Zn^2+^ release curves of PB, ZnPB-1 and ZnPB-3 (200 p.p.m.) at 37 °C in PBS for short-term release (1 day) and long-term release (1 week). Short-term released ions (1 day) are mainly from interstitial site and long-term released ions (1 week) are mainly from lattice site. Error bars indicate means ± standard deviations (*n* = 2 independent samples). Source data are provided as a Source Data file

The cumulative short-term ion release (1 day) and long-term ion release (1 week) in PBS (pH 7.4) at 37 °C, as well as the related mechanism, are shown in Fig. 4. The trend of Fe^2+^ and Fe^3+^ released from 200 p.p.m. PB, ZnPB-1 and ZnPB-3 in PBS (pH 7.4) at 37 °C is similar within 7 days. In general, the release of Fe^2+^, Fe^3+^ and Zn^2+^ is rapid in the first day and slows/stabilizes over the course of a week in PBS (pH 7.4) at 37 °C. Specifically, the cumulatively released Fe^2+^ and Fe^3+^ ions from PB (1.014 ± 0.044 p.p.m. for 1 day and 1.972 ± 0.064 p.p.m. for 1 week) exhibit higher concentrations than those of ZnPB-1 (0.913 ± 0.077 p.p.m. for 1 day and 1.869 ± 0.044 p.p.m. for 1 week) and ZnPB-3 (0.813 ± 0.045 p.p.m. for 1 day and 1.673 ± 0.043 p.p.m. for 1 week), in which Zn^2+^ replaces Fe^2+^ when the former is doped into the lattice sites of PB. Simultaneously, the cumulative release of Zn^2+^ from ZnPB-3 (0.566 ± 0.039 p.p.m. for 1 day and 1.116 ± 0.021 p.p.m. for 1 week) is faster than that from ZnPB-1 (0.350 ± 0.034 p.p.m. for 1 day and 0.933 ± 0.050 p.p.m. for 1 week) due to the increased Zn doping density. In general, the release of Fe^2+^, Fe^3+^ and Zn^2+^ from PB, ZnPB-1 and ZnPB-3 lasts up to a week without initial burst release.

To evaluate the relative stability in PBS, the photothermal heating curves of PB, ZnPB-1 and ZnPB-3 solution irradiated with an 808 nm laser (1.2 W cm^−2^) for 5 min are performed for different periods (0, 1 and 7 days), as shown in Supplementary Fig. 7. Obviously, the photothermal heating curves of PB, ZnPB-1 and ZnPB-3 solutions on day 1 (*n* = 3, *P* > 0.05, compared with day 0) are not significantly different from those on day 0, maintaining the photothermal characteristics in PBS for 1 day. However, the photothermal heating curves of PB, ZnPB-1 and ZnPB-3 solutions on day 7 (*n* = 3, *P* < 0.01) have a slight decrease compared with those on day 0. These results preliminarily demonstrate that PB, ZnPB-1 and ZnPB-3 solutions in PBS for 1 week show slight degradation and relative stability. Additionally, 200 p.p.m. ZnPB-3 solution is completely degraded in sodium hydroxide solution, as shown in Supplementary Fig. 8. The corresponding concentrations of free Fe^2+^/Fe^3+^ and Zn^2+^ are 27.066 ± 0.386 and 21.313 ± 0.043 p.p.m., respectively. In comparison, the cumulative released amounts of Fe^2+^/Fe^3+^ and Zn^2+^ from 200 p.p.m. ZnPB-3 solution in PBS for 1 week are 1.674 ± 0.043 and 1.116 ± 0.021 p.p.m. (Fig. 4), respectively, and the degree of degradation leaching from 200 p.p.m. ZnPB-3 is very low (6.18% and 5.17%). These results adequately demonstrate that ZnPB-3 solution in PBS for 1 week shows slight degradation and relative stability.

Based on the above analysis of the results, the related mechanism of ion release is illustrated in Fig. 4. First, the ions rapidly released after 1 day come primarily from interstitial sites of PB, ZnPB-1 and ZnPB-3, because the porous of MOF can absorb a large number of metal ions, including Fe^2+^, Fe^3+^ and Zn^2+^, which has no significant influence on the structure and properties of the MOF. Afterwards, the ions that are released slowly and stably over a week or even a more extended period are mainly from the lattice sites of PB, ZnPB-1 and ZnPB-3, implying that the MOF exhibits biodegradation behaviour during relatively long-term immersion in PBS. In general, the structure of MOFs (PB, ZnPB-1 and ZnPB-3) determines the unique release behaviour. That is, the porous of MOF is like a container that can store a large number of metal ions (Fe^2+^, Fe^3+^ and Zn^2+^), and the metal ions exhibit rapid release from the interstitial sites. Moreover, the MOFs have relative long-term biodegradability due to release of ions from the lattice sites. Overall, the characteristics of short-term rapid release and long-term stable release have great potential applications in the regulation of bacterial-killing and cell differentiation.

### Antibacterial activity in vitro

The locally increased temperature (more than 50 °C) generated by 808 nm NIR light irradiation can cause lethality through hyperthermia, denaturation of bacterial proteins and irreversible bacterial destruction, eventually killing bacteria^9,10^. Based on the excellent photothermal conversion efficiency of ZnPB as shown in Supplementary Fig. 5, the appropriate PTT for bacterial infection is performed under 808 nm NIR light irradiation (0.3 W cm^−2^) for 15 min (this treatment is marked as L: Light), and corresponding temperature curves are shown in Supplementary Fig. 9. Specifically, the temperature of irradiated ZnPB-3 (200 p.p.m.) is more than 50 °C after 5 min and <55 °C after 15 min, which causes safe (minimal invasion) and effective bacterial destruction.

**Fig. 5:**
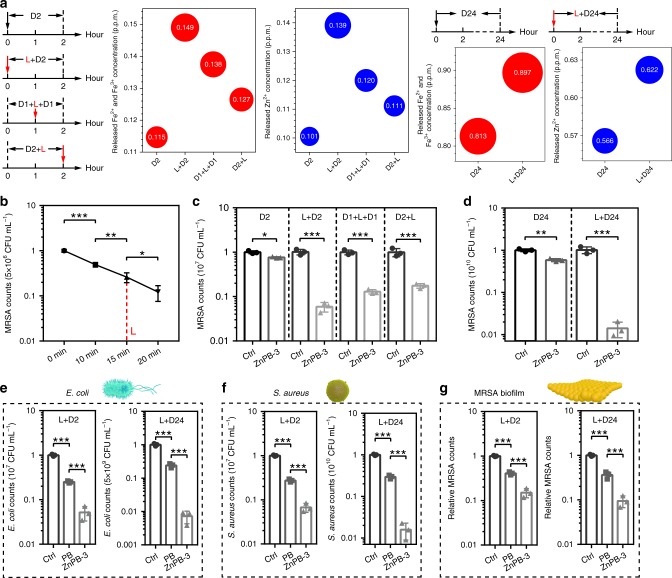
Antibacterial activity in vitro. **a** Schema of giving 808 nm NIR light irradiation at different time point (0, 1 and 2 h) and corresponding amounts of Fe^2+^, Fe^3+^ and Zn^2+^ release after different treatment, including D2, L + D2, D1 + L + D1, D2 + L, D24 and L + D24 (L: light; D: dDark). **b** Kinetics of viability of MRSA treated with ZnPB-3 (200 p.p.m.) by 808 nm NIR light irradiation in 20 min (L: 15 min). **c**, **d** Viability of MRSA treated with ZnPB-3 (200 p.p.m.) through different treatment, including **c** D2, L + D2, D1 + L + D1 and D2 + L, **d** D24 and L + D24 (Ctrl: control). **e**–**g** Viability of MRSA treated with ZnPB-3 (200 p.p.m.) for **e** *E. coli*, **f** *S. aureus* and **g** MRSA biofilm through different treatment (L + D2 and L + D24). Error bars indicate means ± standard deviations (*n* = 3 biologically independent samples): *P < 0.05, **P < 0.01 and ***P < 0.001 (*t* test). Source data are provided as a Source Data file

To comprehensively explore the optimized synergistic antibacterial action of heat and ion release in ZnPB-3 (200 p.p.m.), 808 nm NIR light is utilized to irradiate bacterial medium with 200 p.p.m. ZnPB-3 for 15 min at a specific set point after culturing for 0, 1 and 2 h. The corresponding amounts of Fe^2+^, Fe^3+^ and Zn^2+^ release (L: light; D: dark) are presented in Fig. 5a. Obviously, the amounts of released ions increase in the order D2, D2 + L, D1 + L + D1, and L + D2, demonstrating that the local heat triggered by the photothermal effect will accelerate the release of ions. Moreover, the earlier that local heat is applied, the more ions that will be released. This may be because ZnPB-3 under 808 nm NIR light can rapidly convert light energy to heat energy, and the locally increased heat triggered by the photothermal effect in MOF accelerates the vibration of ions from the interstitial sites of ZnPB-3, thus accelerating the release rates of ions. Similarly, the L + D24 group and D24 group are shown in Fig. 5a (rightmost images), where the amounts of released ions in the L + D24 group are larger than those in the D24 group. Additionally, the antibacterial activity of ZnPB-3 (200 p.p.m.) against MRSA is assessed by different treatments (D2, L + D2, D1 + L + D1, D2 + L, D24, and L + D24), as shown in Fig. 5b, c.

The antibacterial kinetics of ZnPB-3 (200 ppm) with 808 nm NIR light irradiation for 20 min (L represents 15 min) are shown in Fig. 5b; the results indicate that the antibacterial efficiency is time-dependent, and the L group exhibits a moderate antibacterial rate of 73.92 ± 6.42% (*P* < 0.01) against MRSA. Compared with the control group, all the experimental groups show certain antimicrobial efficiencies against MRSA, as seen in Fig. 5c. Specifically, the D2 group shows the lowest antibacterial activity, with antibacterial rates of 23.70 ± 2.68% (*P* < 0.05) against MRSA. The L + D2 group (94.16 ± 1.42%, *P* < 0.001) displays greater bacterial killing efficiency against MRSA than that of the D1 + L + D1 group (87.04 ± 1.44%, *P* < 0.001) and the D2 + L group (82.43 ± 2.06%, *P* < 0.001). To further evaluate the synergistic effect of local heat and the relatively long-term release of ions, the co-incubation time of ZnPB-3 and MRSA is extended to 24 h. The L + D24 group shows the highest antibacterial effects, with an antibacterial rate of 98.61 ± 0.56% (*P* < 0.001) against MRSA, which is far higher than that of the D24 group (41.69 ± 4.89%, *P* < 0.01), as shown in Fig. 5d. The greater bactericidal efficiency can be ascribed to the larger amounts of Zn^2+^, Fe^2+^ and Fe^3+^ released in 24 h than in 2 h (Fig. 5), further confirming the synergistic effect of heat and ion release. For the control (Ctrl, PBS) groups without and with 808 nm light irradiation, the MRSA counts exhibit no significant differences, as shown in Fig. 5c, d. Additionally, when the irradiation time increases from 15 to 20 min (Supplementary Fig. 10A), the corresponding antibacterial rates against MRSA after subsequent culture in dark for 2 and 24 h increase to 99.66 ± 0.15% (2.47-Log reduction, *P* < 0.001, Supplementary Fig. 10B) and 99.99958 ± 0.00029% (5.38-Log reduction, *P* < 0.001, Supplementary Fig. 10C), respectively.

To systematically evaluate the separate antimicrobial efficiency of the corresponding heat and ion release of ZnPB-3, incubation is performed for 2 and 24 h, respectively, in the dark without the effect of ion release after L treatment, where the mixture of treated bacteria and ZnPB-3 is centrifuged and separated with fresh culture medium several times. As shown in Supplementary Fig. 11, for separate incubations for 2 h (72.16 ± 2.67%, *P* < 0.001) and 24 h (69.41 ± 1.72%, *P* < 0.001) in the dark without the effect of ion release after L treatment, a lower bacterial killing efficiency against MRSA is displayed than that for the L + D2 group and L + D24 group, further demonstrating the synergistic antimicrobial efficiency of heat and ion release of ZnPB-3. This enhanced inactivation efficiency by different treatments can be attributed to the fact that the local heat triggered by a photothermal effect not only accelerates the release of ions but also damages the bacterial membrane. That is, the photothermal effect of ZnPB-3 will effectively damage the bacterial membrane and increase its permeability, leading to further permeation of released ions and disturbance of the intracellular metabolic pathways of the bacteria. The related mechanism will be discussed later. Additionally, as shown in Supplementary Fig. 12, the ZnPB-3 group (98.59 ± 0.56%, *P* < 0.05) has a higher antibacterial rate than that of PB (69.46 ± 2.69%, *P* < 0.001), ZnPB-1 (81.18 ± 3.27%, *P* < 0.01) and ZnPB-2 (89.66 ± 2.36%, *P* < 0.05) against MRSA in the L + D24 treatment, demonstrating that ZnPB-3 has the highest antibacterial rate.

The antibacterial activity of ZnPB-3 (200 p.p.m.) is assessed against *Escherichia coli*, *S. aureus*, and MRSA biofilm through different treatments (L + D2 and L + D24). For *E. coli* (Fig. 5d), ZnPB-3 (94.83 ± 1.85% in the L + D2 group and 99.27 ± 0.29% in the L + D24 group) displays dramatically greater bacterial killing efficiency (*P* < 0.001) than that of PB (74.94 ± 1.24% in the L + D2 group and 76.02 ± 3.25% in the L + D24 group). For *S. aureus* (Fig. 5e), the disinfection rates of ZnPB-3 (93.26 ± 1.53% in the L + D2 group and 98.42 ± 0.69% in the L + D24 group) are significantly greater (*P* < 0.001) than that of PB (72.55 ± 2.04% in the L + D2 group and 70.45 ± 3.07% in the L + D24 group). Particularly for the MRSA biofilm, the bacteria are encapsulated in self-produced extracellular polymeric substances, which serve as a protective barrier. Therefore, the bactericidal efficiency for MRSA biofilm eradication is relatively low (84.92 ± 3.55% in the L + D2 group and 90.54 ± 2.79% in the L + D24 group for ZnPB-3; 59.11 ± 3.06% in the L + D2 group and 63.65 ± 6.34% in the L + D24 group for PB, *P* < 0.001), as shown in Fig. 5f. These results further imply that ZnPB-3 has greater antibacterial applications than PB does. In general, this platform can provide a broad-spectrum antibacterial strategy through synergistic heat and ion release.

### Antibacterial mechanism

**Fig. 6:**
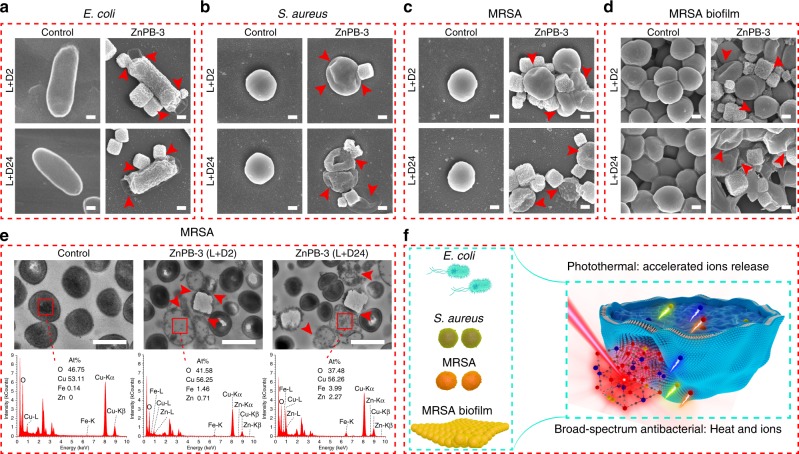
Antibacterial mechanism. SEM morphologies of bacteria, including **a** *E. coli*, **b** *S. aureus*, **c** MRSA and **d** MRSA biofilm, interacted with ZnPB-3 for L + D2 and L + D24 groups (scale bars = 200 nm). **e** TEM images of ultrathin section and corresponding EDS of MRSA treated with ZnPB-3 (scale bars = 1 μm). **f** Antibacterial mechanism of optimized synergistic effect of heat and ion release. Red arrows show severe damage and obvious cytoplasm leakage of bacteria. Source data are provided as a Source Data file

The morphologies of bacteria (*E. coli*, *S. aureus*, MRSA and MRSA biofilm) interacting with ZnPB-3 for the L + D2 and L + D24 groups are qualitatively evaluated by scanning electron microscope (SEM) observation. As shown in Fig. 6a, the morphology of *E. coli* with no treatment (control) is rod-like and unbroken with an integrated membrane, but is irregular, corrugated or completely lysed (red arrow) when treated with ZnPB-3 for the L + D2 and L + D24 groups. Similarly, compared with *S. aureus* and MRSA without treatment, *S. aureus* and MRSA interacting with ZnPB-3 in the L + D2 and L + D24 groups become corrugated and distorted or even partly lysed, as shown in Fig. 6b, c. Moreover, the degree of bacterial destruction in the L + D24 group is more serious than that in the L + D2 group. Even for MRSA biofilm, ZnPB-3 in the L + D2 and L + D24 groups has effective lethality against bacteria. The vast majority of treated bacteria are fused together due to the destruction of their membranes (Fig. 6d).

As shown in Fig. 6e, in the case of the control groups, the transmission electron microscopy (TEM) images show the normal structures of MRSA, that is, regular spherical shapes with intact bacterial membranes. Energy-dispersive spectroscopy (EDS) detection shows no signal of Zn and a small amount of Fe (0.14%) in bacteria. In contrast, the membranes of MRSA interacting with ZnPB-3 are severely damaged and show obvious cytoplasm leakage (Fig. 6e, red arrows), which is caused by the synergistic antibacterial action of heat and ion release. In addition, the EDS spectra show that the Zn and Fe content in bacteria in the L + D24 group (Fe 3.99% and Zn 2.27%) is higher than that in the L + D2 group (Fe 1.46% and Zn 0.71%). Accordingly, the L + D24 group exhibits much higher antibacterial efficacy than the L + D2 group does. That is, the synergistic action of local heat triggered by the photothermal effect and release of Zn^2+^, Fe^2+^ and Fe^3+^ ions plays a key role in bacteria killing.

The mechanism underlying the broad-spectrum bactericidal action of ZnPB-3 is illustrated in Fig. 6f. First, the photothermal effect of ZnPB-3 will effectively damage the bacterial membrane and increase its permeability. The destruction of the bacterial membrane will result in reduced of heat resistance of the cell membrane, leading to further permeation by released ions. Meanwhile, the local heat triggered by the photothermal effect accelerates the release of ions. The released Zn^2+^, Fe^2+^ and Fe^3+^ can rapidly penetrate bacteria, resulting in disturbance of the intracellular metabolic pathways of bacteria and enhancing the bactericidal efficiency. The collaborative action of local photothermy and released ions provides a broad-spectrum antibacterial strategy and brings more insight and understanding of the antibacterial application.

### Cellular morphology, viability and gene expression

Supplementary Figure 13A shows the cell morphology examined by staining with fluorescein isothiocyanate (FITC) and 4′,6-diamidino-2-phenylindole (DAPI) to visualize the F-actin and nuclei, respectively. After incubation for 1 day without 808 nm NIR light irradiation, the cells spread well and exhibit a polygonal morphology with filopodia extensions for all samples. Abundant cellular extension indicates excellent cytocompatibility without appreciable cytotoxicity. In comparison with the control and PB groups, the ZnPB-3 groups (50 and 200 p.p.m.) have the largest number of cells, and most of the cells show a polygonal shape with a large number of filopodia and lamellipodia, suggesting that ZnPB-3 favours the growth and proliferation of cells. In contrast, compared with those in the control group, the cells in the PB and ZnPB-3 groups spread poorly and show a relatively spherical morphology without filopodia extensions under 808 nm NIR light irradiation, suggesting that local heat triggered by the photothermal effect of PB and ZnPB-3 has certain cytotoxicity and lethality to cells.

The viability of cells treated with PB and ZnPB-3 is shown in Supplementary Fig. 13B. The PB and ZnPB-3 groups with concentrations varying from 25 to 200 p.p.m. exhibit excellent cytocompatibility. Specifically, on day 1, the largest cell viability of the 25 ppm ZnPB-3 group is 110.87 ± 9.41% (*P* < 0.05), and the lowest cell viability of the 200 p.p.m. PB group is 85.51 ± 2.95%. In general, PB and ZnPB-3 show satisfactory cytocompatibility without apparent cytotoxicity. After 808 nm NIR light irradiation, the PB and ZnPB-3 groups exhibit definite cell toxicity compared with that of the control group. As the concentration is increased, the cell toxicity of the PB and ZnPB-3 groups gradually increases in comparison with that of the control group due to the higher local heat. The maximum cytotoxicity of the 200 p.p.m. PB and ZnPB-3 groups on day 1 is 45.72 ± 8.93% (*P* < 0.001) and 39.15 ± 8.25% (*P* < 0.001) viability, respectively. However, the cell viabilities of the PB group (53.42 ± 4.18%, *P* < 0.001) and the ZnPB-3 group (60.44 ± 6.19%, *P* < 0.001) on day 3 are much higher than those on day 1, as seen in Supplementary Fig. 14, confirming that the cytotoxicity caused by local heat can be significantly relieved with increasing incubation time.

To understand the related gene expressions (*MMP-2*, *COL-I*, *COL-III*, and *IL-1β*) of tissue repair, NIH-3T3 cells are co-cultured with PB and ZnPB-3 for different periods, and real-time quantitative polymerase chain reaction (RT-qPCR) analyses are performed on days 2, 4 and 8 (Supplementary Fig. 13C). The specific forward and reverse primer sequences of the above-mentioned genes are listed in Supplementary Table 2. Generally, the ZnPB-3 group shows higher upregulation of *MMP-2*, *COL-I* and *COL-III* than that in the PB group when normalized to the expression levels in the control group on day 2, suggesting that released Zn^2+^ from ZnPB-3 plays an important role in regulating the related gene expression. Specifically, in the first 2 days, the relative expression levels of *MMP-2*, *COL-I* and *COL-III* in the PB group and the ZnPB-3 group are slightly upregulated (<2 times) compared with those in the control group. Over time, the relative expression levels of *MMP-2*, *COL-I* and *COL-III* increase significantly in the PB group (1.2–2.5 times) and the ZnPB-3 group (2.0–4.2 times) compared with those in the control group (1.0–1.7 times). Additionally, the relative expression level of *IL-1β* decreases noticeably in the PB group (0.4–0.6 times) and the ZnPB-3 group (0.2–0.8 times) compared with that in the control group (0.6–1.0 times) on days 2, 4 and 8.

*MMP-2* is a member in the family of zinc-dependent and calcium-containing endoproteinases that can be degraded into gelatine in the extracellular matrix (ECM) to promote the migration of fibroblasts^29^. Therefore, the release of Zn^2+^ from ZnPB-3 can improve the chemosynthesis of zinc-based metalloenzymes such as MMPs. Collagen is of significant importance to the proliferative and remodelling phases of the wound repair process, providing a foundation for matrix formation^29^. High expression levels of *COL-I* and *COL-III* in wounds are beneficial for ECM reconstitution due to collagen deposition and wound repair^30^. The released Zn^2+^ from ZnPB-3 can improve the expressions of *COL-I* and *COL-III*, thus promoting collagen deposition. *IL-1β* is an inflammatory factor that hinders the recovery of wounds^29^. Moreover, Zn^2+^ acts as an anti-inflammatory antioxidant that can downregulate pro-inflammatory signals by reducing the release of inflammatory mediators^29^. Therefore, the released Zn^2+^ from ZnPB-3 suppresses the expression of *IL-1β* to reduce the inflammatory response. In summary, the upregulated gene expression (*MMP-2*, *COL-I* and *COL-III*) and downregulated gene expression (*IL-1β*) due to ZnPB-3 group can improve the chemosynthesis of MMPs, promote collagen deposition and inhibit inflammatory factors to favour wound repair.

### In vivo PTT for MRSA wound infections

**Fig. 7:**
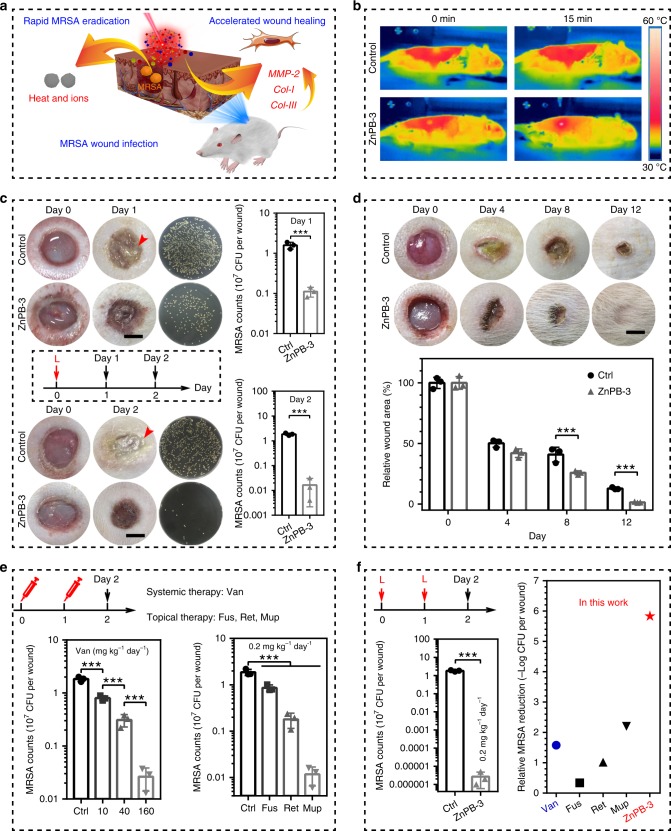
In vivo photothermal therapy for MRSA wound infections. **a** Mechanism of photothermal therapy of ZnPB-3 for MRSA wound infections in vivo. **b** Photothermal images of control and ZnPB-3 groups during NIR irradiation. **c** Photographs of MRSA-infected wounds (scale bars = 3 mm). Red arrows mark serious suppuration on the wounds; quantitative assessment of bacterial counts in wounds by spread plate method and corresponding viability of MRSA on days 1 and 2. **d** Representative photographs of the wound healing process and corresponding quantitative analysis of wounds over time (scale bars = 3 mm). **e** Viability of MRSA treated by Van with different dosage (10, 40 and 160 mg kg^−1^ day^−1^) on day 2 in vivo; viability of MRSA treated with Fus, Ret and Mup using same dosage (0.2 mg kg^−1^ day^−1^) on day 2 in vivo. **f** Viability of MRSA treated with ZnPB-3 (0.2 mg kg^−1^ day^−1^) on day 2 in vivo; comparison of therapeutic effects among Van (160 mg kg^−1^ day^−1^), Fus, Ret, Mup and ZnPB-3 using same dosage (0.2 mg kg^−1^ day^−1^) on day 2 in vivo. Error bars indicate means ± standard deviations (*n* = 3 biologically independent samples): ***P < 0.001 (*t* test). Source data are provided as a Source Data file

Based on the encouraging results of the above-described antibacterial mechanism, the in vivo therapeutic efficacy of ZnPB-3 is evaluated using an animal MRSA wound model, and the related scheme is shown in Fig. 7a. The synergistic antibacterial action of heat and ion release from ZnPB-3 can rapidly eradicate MRSA in wounds. To balance the antimicrobial efficiency and toxicity to healthy tissue during PTT, an appropriate range of temperatures for PTT with ZnPB-3 for 15 min is explored in Supplementary Fig. 15, and the corresponding antimicrobial efficiency and toxicity to healthy tissue are evaluated. Specifically, the antimicrobial ratio is more than 90% (*P* < 0.001) when the maximum temperature for PTT is more than 55 °C after 15 min. However, the obvious toxicity to healthy tissue can be observed by the corresponding photographs (yellow arrows mark damaged tissue), and the inflammatory response can be observed by in hematoxylin and eosin (H&E) staining (red arrows mark lobulated neutrophils) due to hyperthermia, when the maximum temperature for PTT is more than 60 °C after 15 min. Based on the above results, the appropriate temperature (maximum is ~55 °C) is chosen for subsequent PTT evaluations. Based on the high photothermal conversion efficiency of ZnPB-3 in vitro, PTT for MRSA wound infection in vivo is carried out as shown in Fig. 7b. MRSA-infected rats are anaesthetized and exposed to an 808-nm NIR laser at a power density of 0.3 W cm^−2^, which is within the scope of the maximum permissible exposure for skin exposure formulated by the American National Standards Institute (ANSI Z136.1-2000), and the infected wound-site temperature rapidly increases from ∼35 °C to ∼55 °C over 15 min of laser irradiation. In comparison, the temperature of the control group under only 808 nm NIR laser irradiation shows no significant change.

At 1 and 2 days post infection, the control groups show an obvious inflammatory response and serious suppuration of the wounds (red arrows in Fig. 7c) due to severe bacterial burdens in the wounds. In contrast, ZnPB-3 treatment results in no observed inflammatory response and complete escharosis, preliminarily indicating that the ZnPB-3 groups can sterilize MRSA-infected wounds in a rat model. Additionally, the photothermal treatment has no obvious influence on the healthy skin because the localized temperature is <55 °C, and the bacterial counts in MRSA-infected wounds are quantitatively assessed using the spread plate method. The MRSA counts show no significant differences (*P* > 0.05, Supplementary Fig. 16) in the control (Ctrl, PBS) groups without and with 808 nm light irradiation for 15 min with subsequent culture in the dark for 1 day and 2 days, respectively. Obviously, the ZnPB-3 groups (0.2 mg kg^−1^) display excellent bacterial killing efficiency against MRSA on day 1 (92.95 ± 1.93%, *P* < 0.001) and day 2 (99.11 ± 0.77%, *P* < 0.001) in vivo, which is consistent with the antibacterial activity in vitro (Fig. 5). In general, the negligible levels of bacteria counts and the decreased wound sizes in the ZnPB-3 groups demonstrate efficacious bacterial elimination and accelerated wound healing.

Representative photographs of the wound healing process exhibit significant differences between the ZnPB-3 groups and control groups in Fig. 7d. The control groups exhibit a certain degree of pyosis on day 4. In contrast, the ZnPB-3 groups clearly show accelerated wound closure compared with that of the control groups, demonstrating that the ZnPB-3 groups can significantly promote wound healing in MRSA infections. The quantitative analysis of the above images of wounds over time also shows that the wound closure rate of the ZnPB-3 groups is significantly faster than that of the control groups (*P* < 0.001).

Vancomycin (Van) is the gold standard for current clinical MRSA treatment^31,32^. Therefore, treatment with Van is selected as a positive control for therapy of MRSA-infected wounds. In Fig. 7e, the Van treatment (minimum inhibitory concentration (MIC) = 1 μg mL^−1^) with different dosages (10, 40 and 160 mg kg^−1^ day^−1^) through intravenous injection shows concentration-dependent antibacterial rates (56.20 ± 4.61% for 10 mg kg^−1^ day^−1^, 83.09 ± 4.46% for 40 mg kg^−1^ day^−1^, and 98.56 ± 0.67% for 160 mg kg^−1^ day^−1^, *P* < 0.001) against MRSA infection on day 2. However, as a systemic antibiotic therapy, the systemic toxicity of Van at high concentrations (160 mg kg^−1^ day^−1^) is evaluated on day 7, as shown in Supplementary Fig. 17. At high Van concentrations (160 mg kg^−1^ day^−1^), the biochemical measures of renal function are significantly higher (*P* < 0.001) than those of the corresponding control, including blood urea nitrogen (BUN), creatinine (CR) and uric acid (UA), indicating certain nephrotoxicity^33^. Additionally, although Van is typically used as the last resort, Van-resistant pathogens are now widespread^34,35^.

Fusidic acid (Fus, MIC = 128 μg mL^−1^), retapamulin (Ret, MIC = 0.5 μg mL^−1^), and mupirocin (Mup, MIC = 0.5 μg mL^−1^) are selected as a positive control for topical antibiotic therapy for MRSA-infected wound^36–39^. The corresponding antibacterial rates of Fus (54.06 ± 7.08%, *P* < 0.001), Ret (90.40 ± 3.46%, *P* < 0.001), and Mup (99.38 ± 0.27%, *P* < 0.001) using the same dosage (0.2 mg kg^−1^ day^−1^) are <99.9% on day 2 in vivo.

Comparatively, the corresponding antibacterial rate of ZnPB-3 (0.2 mg kg^−1^ day^−1^) by irradiation twice against MRSA on day 2 increases to 99.99985 ± 0.00012% (5.83-Log reduction, *P* < 0.001), as shown in Fig. 7f. Therefore, the topical therapy (5.83-Log reduction) in this work against MRSA wound infection shows much higher antibacterial efficiency than that of the current clinical MRSA treatments, including systemic antibiotic therapy (Van) and topical antibiotic therapy (Ret, Fus and Mup). Additionally, the faster antibacterial efficiency in this work relative to these antibiotic therapies is shown in Supplementary Fig. 18, where the therapy using ZnPB-3 in this work can kill most of the MRSA effectively in 15 min, in contrast, the other antibiotic therapies show no antibacterial effects.

### Histomorphological examination of the wound healing

Many lobulated neutrophils from H&E staining (red arrows in Supplementary Fig. 19A) can be observed in the wounds in the control groups, indicating severe bacterial infection. A large number of neutrophils in wounds demonstrate severe bacterial infection because neutrophils will migrate rapidly from circulating blood to infected sites^40^. In comparison, the number of neutrophils in the ZnPB-3 groups is lower than that in the control groups, illustrating the relatively minor infection and effective antibacterial ability of ZnPB-3 in vivo.

Additionally, Masson’s trichrome staining and Sirius red staining reflect more new collagen deposition (blue colour for Masson’s trichrome staining and red colour for Sirius red staining) in the wounds on day 12, as shown in Supplementary Fig. 19B. Obviously, the ZnPB-3 groups have significantly higher levels of collagen deposition than the control groups do. To further evaluate collagen accumulation within the wound, the quantitatively relative areas of collagen in the Masson’s trichrome staining and Sirius red staining (corresponding different higher magnification in Supplementary Fig. 19B) are shown in Supplementary Fig. 19C, D. Similarly, the ratio of collagen-occupied regions of the ZnPB-3 groups is higher than that of the control groups (*P* < 0.01), which is consistent with the results shown in Supplementary Fig. 13C.

### In vivo toxicology

The in vivo toxicology is evaluated by the corresponding histological analyses of major organs (liver, spleen, kidney, heart and lung) through H&E staining on day 12, as shown in Supplementary Fig. 20. No significant differences and no sign of organ damage between the control and ZnPB-3 are observed, suggesting that ZnPB-3 has no apparent histological toxicology in vivo. As shown in Supplementary Fig. 21, the standard haematology data between the control and ZnPB-3 groups on day 12 exhibit no significant differences (*P* > 0.05) and each of the measure is within the corresponding normal scope (marked by dashed lines), including haematocrit, haemoglobin (HGB), **mean corpuscular** **H**GB concentration, mean corpuscular haemoglobin, mean corpuscular volume, mean platelet volume, platelet count, red blood cell count, red cell distribution width and white blood cell count. Further, biochemical analyses of hepatic function (Supplementary Fig. 22A) and renal function (Supplementary Fig. 22B) show no significant differences (*P* > 0.05) between the control and ZnPB-3 groups on day 12, including alkaline phosphatase, alanine transaminase, aspartate transaminase, blood urea nitrogen, creatinine and uric acid. In addition, the concentrations of ions (Fe^2+^, Fe^3+^ and Zn^2+^) in blood (Supplementary Fig. 22C) and urine (Supplementary Fig. 22D) show no significant differences (*P* > 0.05) between the control and ZnPB-3 groups on day 12. According to the above results, ZnPB-3 shows excellent biosafety. In summary, the advantages in our work over the current clinical MRSA treatments are as follows: (1) faster and higher antibacterial efficiency; (2) lower dosage; and (3) no systemic toxicity.

## Discussion

ZnPB with various doping levels is controllably designed to enhance its photothermal property for combating MRSA wound infection through the optimized synergistic antibacterial effect of heat and ion release. The mechanism of enhanced photothermal conversion efficiency of ZnPB is ascribed to the bandgap-narrowing effect and the LSPR moving towards lower energy with increasing Zn doping density. Simultaneously, the relative long-term biodegradability of ZnPB is illustrated by the characteristics of short-term rapid release from the interstitial sites and long-term stable release from the lattice sites. The mechanism underlying the broad-spectrum bactericidal action of ZnPB-3 originates from synergistic local heat and released ions. The in vivo results confirm the relatively minor MRSA infection and effective antibacterial ability of ZnPB-3, and the released Zn^2+^ from ZnPB-3 can improve collagen deposition to favour wound healing. Controllable regulation of a photonic MOF for combating MRSA wound infection provides a valuable alternative to current antibiotic therapies in the post-antibiotic era. Moreover, this platform will bring further insight and understanding of multidisciplinary applications not only in the treatment of MRSA infection but also in wound repair and the design of photonic MOFs.

## Methods

### Theoretical calculations

DFT calculations were carried out by VASP. First-principles electronic structure calculations were performed within the generalized gradient approximation (GGA) in the Perdew-Burke-Ernzerhof form. The interactions between ions and electrons were represented by the projector-augmented wave method with a cutoff energy of 600 eV. Uniform G-centred k-point meshes with a resolution of 2π*0.03 Å^-1^ and Methfessel-Paxton electronic smearing were used for integration in the Brillouin zone. These settings assured convergence of the total energies to within 1 meV per atom. Structure relaxation proceeded until all forces on the atoms were less than 1 meV Å^-1^ and the total stress tensor was within 0.01 GPa of the target value.

### Materials

Potassium ferricyanide (K_3_[Fe(CN)_6_]), zinc chloride (ZnCl_2_), Van hydrochloride, and Mup were purchased from Aladdin (Shanghai, China). Poly(vinylpyrrolidone) (PVP, K30) and hydrochloric acid (HCl, 36.0%-38.0%) were purchased from Sinopharm Chemical Reagent Co., Ltd. Fus sodium salt was purchased from TargetMol (China), and Ret was purchased from Yuanye (China). All chemicals were used directly without further purification.

### Preparation of PB nanocubes

K_3_[Fe(CN)_6_] (0.8 mmol) was dissolved in 80 mL deionized water, and then PVP (6 g) was added to the solution under magnetic stirring to obtain a clear solution. Next, aqueous HCl (0.01 M, 80 mL) was mixed with the above clear solution under magnetic stirring. The mixture was then stored at 80 °C for 20 h. After the reaction, the precipitates were collected by centrifugation and washed three times each with ethanol and deionized water. The synthesized samples were collected after freeze-drying for further use.

### Preparation of ZnPB nanocubes with various doping levels

The synthesis of ZnPB nanocubes was similar to that of PB nanocubes except for the addition of Zn. Typically, K_3_[Fe(CN)_6_] (0.8 mmol) and PVP (3 g) were dissolved in 40 mL deionized water under magnetic stirring to obtain solution A. Various amounts of ZnCl_2_ (0.2, 0.4 and 0.8 mmol) and PVP (3 g) were dissolved in 40 mL deionized water under magnetic stirring to obtain solution B. Then, solution B was slowly mixed with solution A under magnetic stirring until a clear solution was formed. Afterwards, the aqueous HCl (0.01 M, 80 mL) was added. Finally, the mixture solution was kept at 80 °C for 20 h. After the reaction, the precipitates were collected by centrifugation and washed three times each with ethanol and deionized water. The products were obtained after freeze-drying.

### Characterization of morphology and structure

A high-resolution transmission electron microscopy (Talos F200S) system equipped with EDS was employed to observe the microstructure of the samples. The crystal structure of the samples was investigated by XRD (D/MAX-IIIC, Rigaku). The UV–Vis–NIR absorption between 300 nm and 1000 nm was measured on a microplate reader (SpectraMax I3MD USA). The corresponding bandgap was calculated based on UV–Vis–NIR absorption determined by a UV–Vis–NIR spectrometer (UV–Vis–NIR, UV-3600, Shimadu, Japan).

### Photothermal performance measurements

The temperature trends of different concentrations (25, 50, 100, and 200 p.p.m.) of PB and ZnPB solution in PBS were measured under irradiation with an 808 nm laser (Hi-Tech Optoelectronics Co., Ltd., China). Specifically, 0.5 mL PB and ZnPB solutions in PBS in an EP tube (Eppendorf tube) were irradiated with 808 nm NIR laser (1.2 W cm^−2^) for 5 min. The control group was pure PBS. The temperature of the samples was recorded using real-time thermal imaging monitored by a FLIR thermal camera (FLIR-E64501, Estonia). The calculation of the photothermal conversion efficiency (*η*) of PB and ZnPB can be obtained by the following Eq. (1):

1 $$\eta = \frac{{hS(T_{\max } - T_{{\mathrm{amb}}}) - Q_0}}{{I(1 - 10^{ - A})}},$$

where *h* represents the heat transfer coefficient, *S* is the surface area of the container, *T*_max_ is the equilibrium temperature, *T*_amb_ represents the surrounding ambient temperature, *Q*_0_ is the heat absorption of EP tube (Eppendorf tube), *I* represents the laser power and *A* is the absorbance of PB and ZnPB at 808 nm.

If the heat input of the system is equal to the heat output,

2 $$hS = \frac{{\mathop {\sum}\nolimits_i^{} {m_iC_{p,i}} }}{{\tau _S}} \approx \frac{{m_{{\mathrm{H}}_2{\mathrm{O}}}C_{{\mathrm{H}}_2{\mathrm{O}}}}}{{\tau _S}},$$

where $m_{{\mathrm{H}}_2{\mathrm{O}}}$ represents the weight of water, $C_{{\mathrm{H}}_2{\mathrm{O}}}$ is the specific heat capacity of water and *τ*_*S*_ represents the time constant of PB and ZnPB. During the cooling period,

3 $$t = - \tau _S\,\ln\,\theta = - \tau _S\,\ln \frac{{T - T_{{\mathrm{amb}}}}}{{T_{\max } - T_{{\mathrm{amb}}}}}.$$

Therefore, *τ*_*S*_ can be calculated using the linear regression curve through the above Eq. (3)^23^.

### Fe^2+^, Fe^3+^ and Zn^2+^ release experiments

The release behaviour of Fe^2+^, Fe^3+^ and Zn^2+^ from PB (200 p.p.m.) and ZnPB (200 p.p.m.) was investigated in PBS (pH 7.4) at 37 °C at different time points (2 h, 12 h, 1 day, 3 days, 5 days and 7 days). The released Fe^2+^, Fe^3+^ and Zn^2+^ ions were detected by inductively coupled plasma atomic emission spectrometry (Optimal 8000, Perkin-Elmer, USA), inductively coupled plasma optical emission spectrometry (Agilent 700 Series) and inductively coupled plasma mass spectrometry (Agilent 7800 Series), respectively.

### In vitro antibacterial experiments

The in vitro antibacterial activity of PB and ZnPB against Gram-positive MRSA (CCTCC 16465) and *S. aureus* (ATCC 25923) and against Gram-negative *E. coli* (ATCC 8099) was quantitatively assessed by the spread plate method. The bacteria were cultured in standard Luria–Bertani (LB) culture medium. Then, 200 μL bacterial suspensions (5 × 10^6^ colony-forming unit (CFU) mL^−1^) were incubated with PBS (control), PB (200 p.p.m.) and ZnPB-3 (200 p.p.m.) in a 96-well plate without and with 808 nm laser irradiation at a power density of 0.3 W cm^−2^ for 15 min. After various treatments, 20 μL of the appropriate diluted bacterial solution was plated on standard LB agar and incubated at 37 °C for another 24 h. The bacterial colonies were counted and the antibacterial rate was assessed by the following Eq. (4):

4 $${\mathrm{Antibacterial}}\;{\mathrm{ratio}}\;{\mathrm{ }}(\% ) = \frac{{{C} - {E}}}{{C}} 100\%,$$

where *C* and *E* represent the numbers of bacteria (CFUs) in the control group and in the experimental group, respectively.

The morphologies of bacteria (*E. coli*, *S. aureus*, MRSA and MRSA biofilm) interacting with ZnPB-3 by various treatments were qualitatively evaluated by SEM observation. The treated bacteria were fixed with a 2.5% glutaraldehyde solution for 2 h and rinsed twice with sterile PBS. Then, the samples were sequentially dehydrated in ethanol solution with different concentrations (30, 50, 70, 90 and 100%, v/v) for 15 min. The samples were air-dried overnight before SEM observation.

For TEM observation of the inner structures, the bacteria that interacted with ZnPB-3 were collected and centrifuged at 10,000 r.p.m. for 5 min. The samples were fixed with 2.5% glutaraldehyde solution for 2 h and washed with PBS (pH 7.4) three times. Then, the samples were post-fixed in 1% osmic acid at 4 °C for 2 h, and subsequently rinsed with PBS three times. The samples were dehydrated separately in ethanol solution with increased concentrations (50%, 70%, 80%, 90% and 95% for 15 min each and then 100% for 15 min twice). Afterwards, the samples were permeated in the embedding medium (acetone: Epon 812 (SPI 90529-77-4) = 1:1) for 2 h, in the embedding medium (acetone: Epon 812 (SPI 90529-77-4) = 1:2) overnight and in pure Epon 812 overnight at 37 °C. Then, the samples were embedded at 60 °C for 48 h. Ultrathin sections (60–80 nm) were obtained with a microtome (Leica UC7), and then stained with uranylacetate. Finally, TEM sections were dried and prepared before TEM observation.

### In vitro cytocompatibility evaluation

NIH-3T3 (mouse embryonic fibroblast, ATCC CRL-1658) was cultured in minimum essential medium/Earle’s balanced salts medium (HyClone) supplemented with 10% foetal bovine serum, 1% penicillin–streptomycin solution (HyClone) and 1% amino acid under incubation at 37 °C in 5% CO_2_ and 95% humidity. The culture medium was refreshed every 3 days.

For fluorescence morphology, NIH-3T3 cells were first incubated in 96-well plates at 37 °C for 24 h and further incubated with PB and ZnPB at various concentrations (25, 50, 100 and 200 p.p.m.) without and with 808 nm laser irradiation at a power density of 0.3 W cm^−2^ for 15 min. The treated cells were cultured for another 24 h. After incubation, the samples were washed with PBS three times and fixed in 4% formaldehyde for 10 min at room temperature, followed by rinsing with PBS. Then, the samples were stained with FITC; YiSen, Shanghai) for 30 min in the dark, rinsed with PBS three times, and further stained with DAPI (YiSen, Shanghai) for 30 s, and rinsed with PBS three times. The cytoskeletal actin (green fluorescence) stained by FITC and the cell nuclei (blue fluorescence) stained by DAPI were photographed by fluorescence microscopy (IFM, Olympus, IX73).

For the in vitro cytotoxicity assay, NIH-3T3 cells were first seeded in 96-well plates for 24 h. Then, PB and ZnPB at various concentrations (25, 50, 100 and 200 p.p.m) were added to 96-well plates without and with 808 nm laser irradiation at a power density of 0.3 W cm^−2^ for 15 min, and co-cultured with for another 24 h. The control group was pure PBS. Standard MTT (3-(4,5-dimethylthiazol-2-yl)-2,5-diphenyltetrazolium bromide) assays were performed to measure the relative cell activities. After incubation for certain periods, the medium was removed and 0.5 mg mL^−1^ MTT (Aladdin Reagent Co., China) was added, followed by incubation for 4 h at 37 °C. Then, the MTT solution was removed, and 200 μL dimethyl sulfoxide was added to dissolve the crystals under vibration for 15 min. The 96-well plates were centrifuged at 500 r.p.m. for 5 min. Finally, the absorption of the supernatant at 570 nm was determined on a microplate reader (SpectraMax I3MD, USA). The cell viability (%) was calculated by comparing the absorbance values of the samples with those of the control.

### Quantitative real-time reverse transcription polymerase chain reaction

The expression of genes related to tissue repair was evaluated by RT-qPCR. First, NIH-3T3 cells were seeded on 12-well plates for 24 h, and then incubated with PBS (control), PB (200 p.p.m.), and ZnPB-3 (200 p.p.m.) for 2, 4 and 8 days, separately. Afterwards, the cellular total RNA of cells was extracted by a Total RNA Kit (Omega) and reverse transcribed into complementary DNA (cDNA) using PrimeScript RT Master Mix (TaKaRa, Japan) according to the manufacturer’s instructions. The resulting cDNA was used as a template for RT-qPCR with target primers for *IL-1β*, *MMP-2*, *COL-I*, *COL-III* and *β-actin*. The CFX Connect™ Real-Time PCR Detection System (Bio-Rad) was used to conduct real-time fluorescence quantification. The gene expression levels of *IL-1β*, *MMP-2*, *COL-I* and *COL-III* were analysed and the gene expression level of *β-actin* was used for normalization. The specific forward and reverse primer sequences of the above-mentioned genes are listed in Supplementary Table 2. Quantification of gene expression was based on the comparative cycle-threshold method.

### Rat cutaneous MRSA wound infection model

All animal experiments and procedures were approved by Hubei Provincial Center for Disease Prevention and Control, and the Department of Orthopedics, Union Hospital, Tongji Medical College, Huazhong University of Science and Technology, Wuhan, China. All animals were kept and utilized in accordance with the Animal Management Rules of the Ministry of Health of the People’s Republic of China and the Guidelines for the Care and Use of Laboratory Animals of China. Eight-week-old Sprague–Dawley male rats (~200 g) were obtained from the Hubei Provincial Center for Disease Prevention and Control. A total of 120 Sprague–Dawley male rats were randomly divided into the following groups: control, ZnPB-3, Van, Fus, Ret and Mup. First, the rats were anaesthetized by intraperitoneal injection of 3% pentobarbital (1 mL kg^−1^) prior to surgery and placed on a sterile drape to provide sterile conditions during surgery. The dorsal side of the rats was shaved, depilated and disinfected. Two symmetrical round wounds with 6 mm in diameter were made on the dorsum of each rat using a biopsy punch. Then, each wound from each group was infected with a MRSA dose of 10^7^ CFU per wound, and treated with PBS (control) and ZnPB-3 (0.2 mg kg^−1^) with 808 nm laser irradiation at a power density of 0.3 W cm^−2^ for 15 min. Afterwards, the treated wounds were covered with nonwoven fabrics and fixed with surgical adhesive. Comparatively, the treatment with Van is set as the positive control for systemic antibiotic therapy of MRSA-infected wounds using different dosages (10, 40 and 160 mg kg^−1^ day^−1^) through intravenous injection, and the treatments with Van, Fus, Ret and Mup are also set as positive controls for topical antibiotic therapy of MRSA-infected wounds using the same dosage (0.2 mg kg^−1^ day^−1^).

To evaluate the therapeutic effect of ZnPB for MRSA wound infection, the bacterial counts in each wound were assessed by the spread plate method at each time point (days 1 and 2), and the wounds were observed and photographed at regular time intervals. For histomorphological analysis, the rats were sacrificed, and the skin tissues of the wounds were harvested and fixed with 4% paraformaldehyde solution and then embedded in paraffin. The samples were stained with H&E staining on days 2, 4, 8 and 12, Masson’s trichrome staining on day 12 and Sirius red staining on day 12. To evaluate in vivo biosafety, the major organs (heart, liver, spleen, lung and kidney) were stained with H&E on day 12.

### Statistical analysis

All the quantitative data in each experiment were evaluated and analysed by one-way analysis of variance and expressed as the mean values ± standard deviations. Student’s *t* test was used to evaluate the statistical significance of the variance. Values of *P < 0.05, **P < 0.01 and ***P < 0.001 were considered statistically significant.

### Reporting summary

Further information on research design is available in the Nature Research Reporting Summary linked to this article.

## Supplementary information

Supplementary Information

Description of Additional Supplementary Files

Supplementary Data 1

Reporting Summary

Source Data

## Data Availability

Data underlying Figs. 2G, 2H, 3A–3D, 4, 5B–5G, 6E, 7C–7F, Supp Fig. 4–9, 10B, 10 C, 11, 12, 13B, 14–18, 19 C, 19D, 21, 22 are provided in the Source Data File. All other data are available from the corresponding author upon reasonable requests.
