# Supplementary material for: Zinc-doped Prussian blue enhances photothermal clearance of Staphylococcus aureus and promotes tissue repair in infected wounds
Source: Nat Commun. 2019 Oct 3;10:4490. doi: 10.1038/s41467-019-12429-6 (PMC6776522; doi:10.1038/s41467-019-12429-6)
Supplement: Supplementary file 1 — Supplementary Information [file 41467_2019_12429_MOESM1_ESM.doc]

Supplementary Information

**Zinc-doped Prussian blue enhances photothermal clearance of *Staphylococcus aureus* and promotes tissue repair in infected wounds**

Li et al.

**Supplementary Figures**


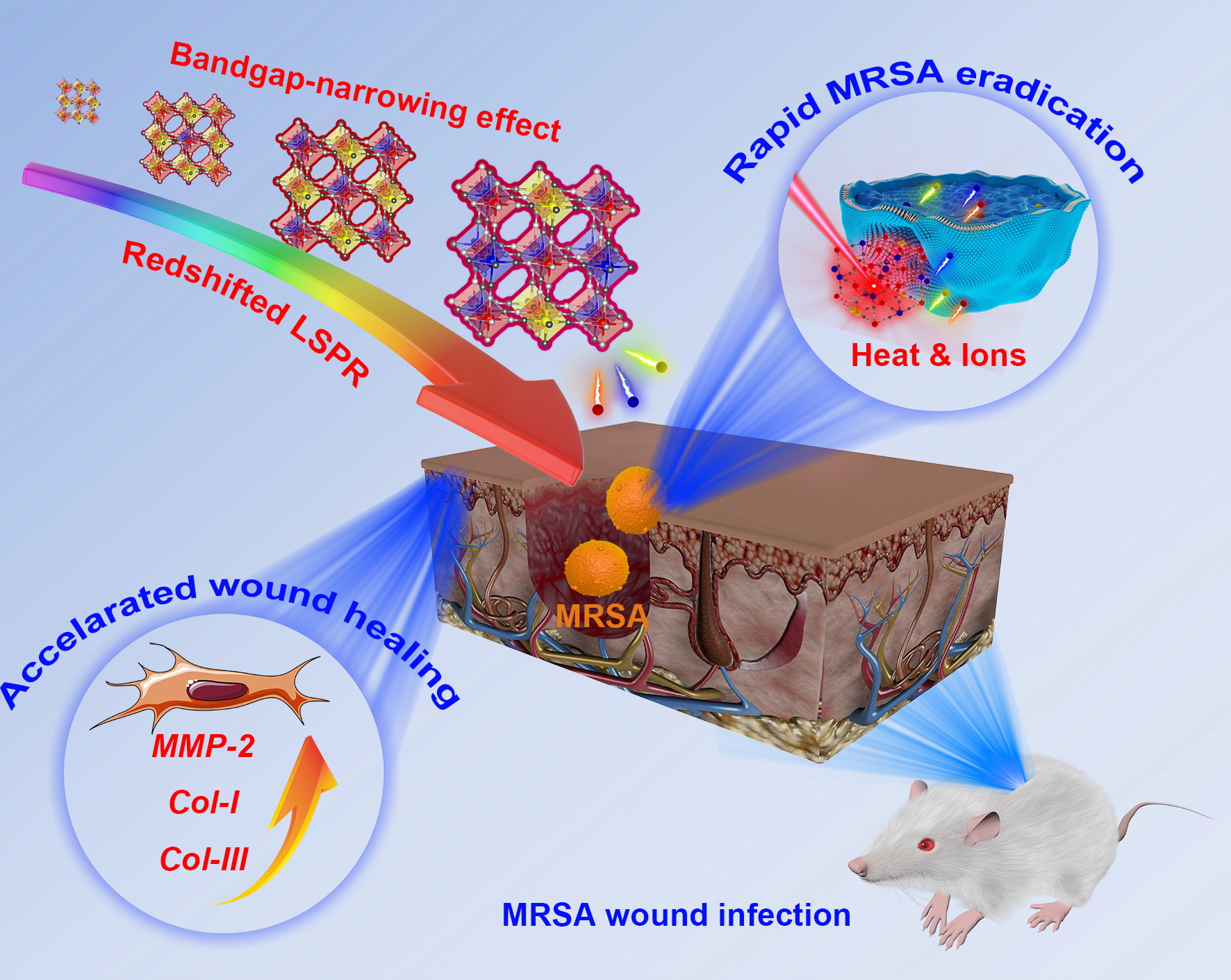


**Supplementary Fig. 1.** Schematic illustration of the controllable regulation of ZnPB with various doping levels to enhance its photothermal property for combating MRSA wound infection through optimized synergistic antibacterial effect of heat and ions.


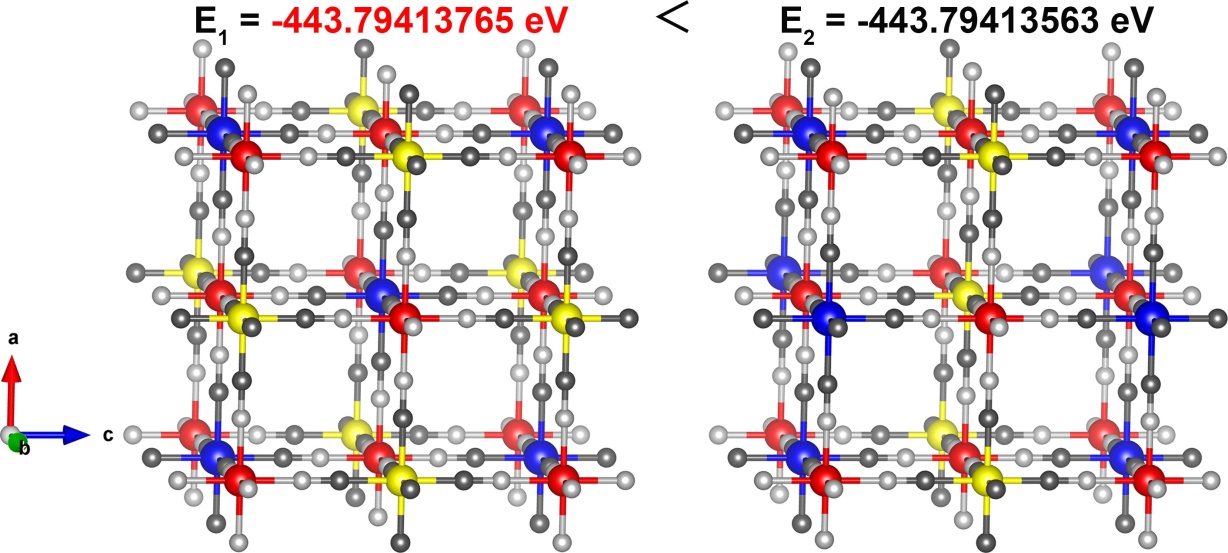


**Supplementary Fig. 2.** Relative stable geometrical structures of ZnPB-2 and corresponding total energy of each unit cell.


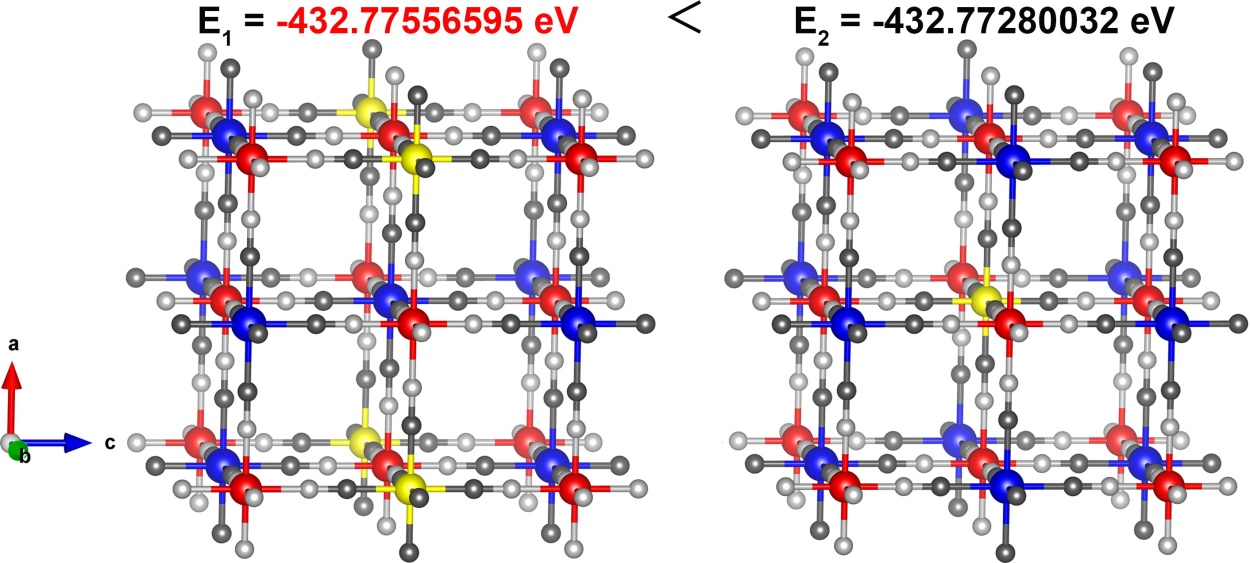


**Supplementary Fig. 3.** Relative stable geometrical structures of ZnPB-3 and corresponding total energy of each unit cell.


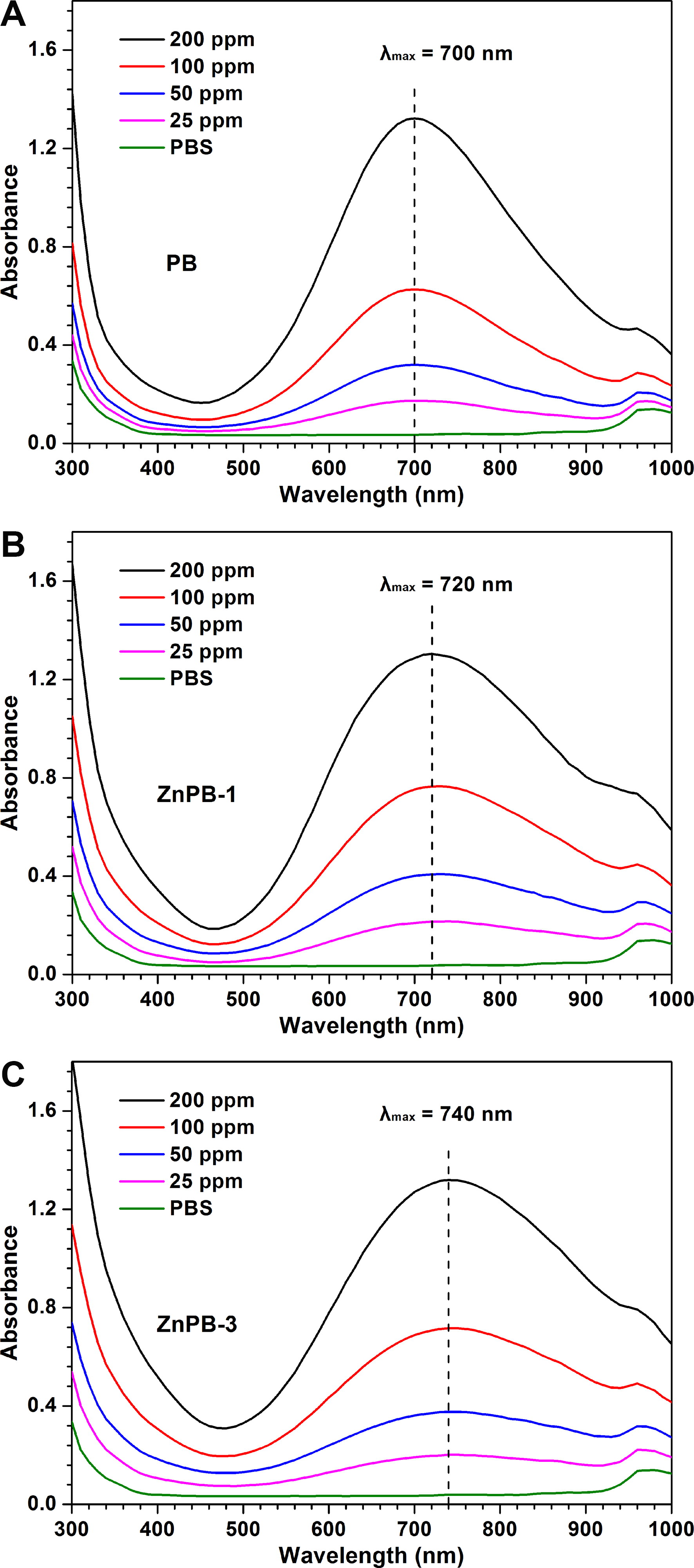


**Supplementary Fig. 4.** Absorption spectra (300-1000 nm) of different concentrations of PB, ZnPB-1, and ZnPB-3. Source data are provided as a Source Data file.


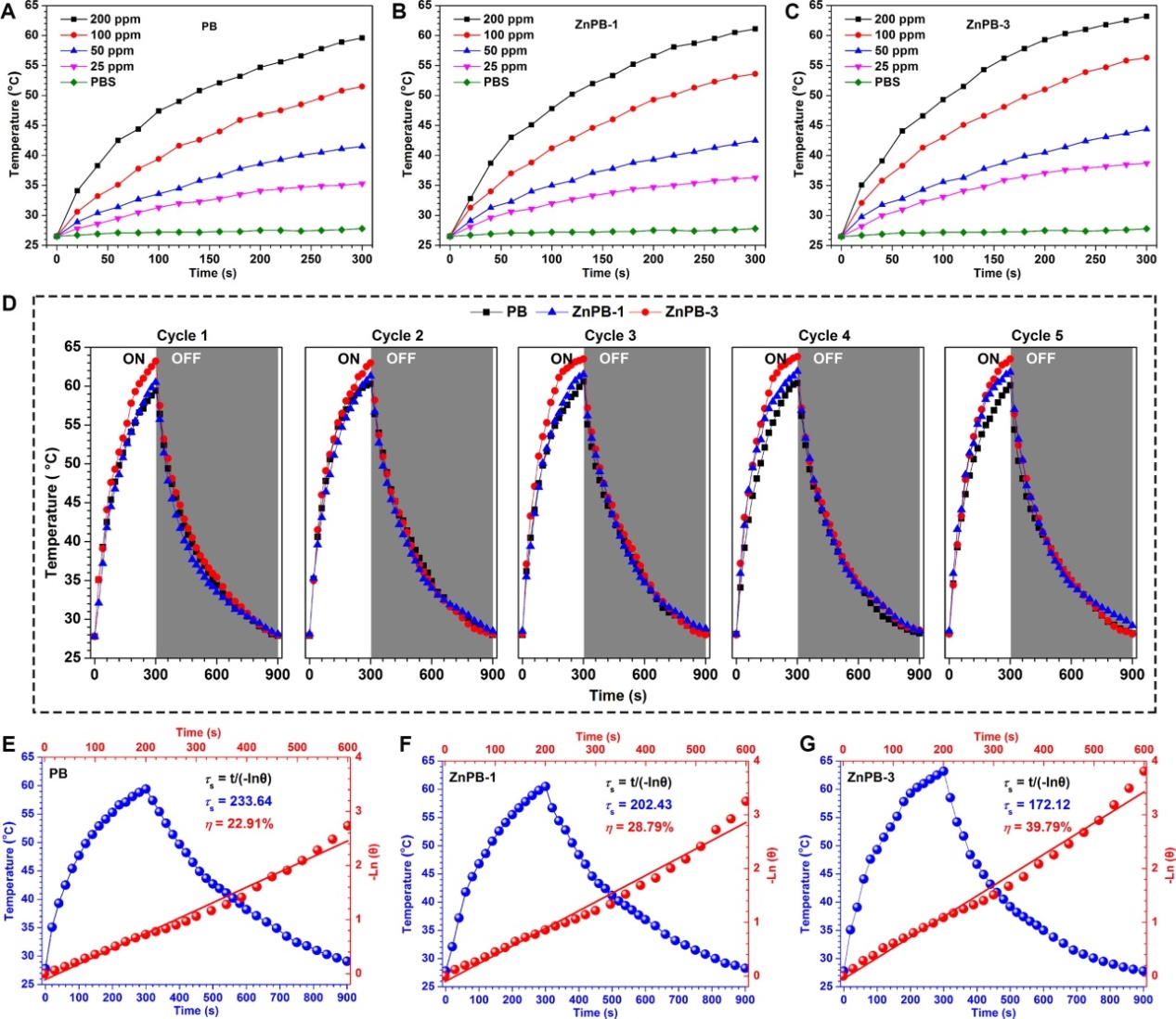


**Supplementary Fig. 5.** Photothermal heating curves of **(A)** PB, **(B)** ZnPB-1, and **(C)** ZnPB-3 with various concentrations (25, 50, 100, and 200 ppm). **(D)** Recycling heating profiles of PB, ZnPB-1 and ZnPB-3 solutions at 200 ppm under 808 nm laser irradiation (1.2 W cm^-2^) for five on/off cycles. Calculation of the photothermal conversion efficiencies (*η*) of **(E)** PB, **(F)** ZnPB-1 and **(G)** ZnPB-3 at 808 nm. Blue line: photothermal effect of PB, ZnPB-1 and ZnPB-3 for certain periods, and then the laser is turned off. Red line: time constant (*τ_s_*) from the cooling period by utilizing the linear time data. Source data are provided as a Source Data file.


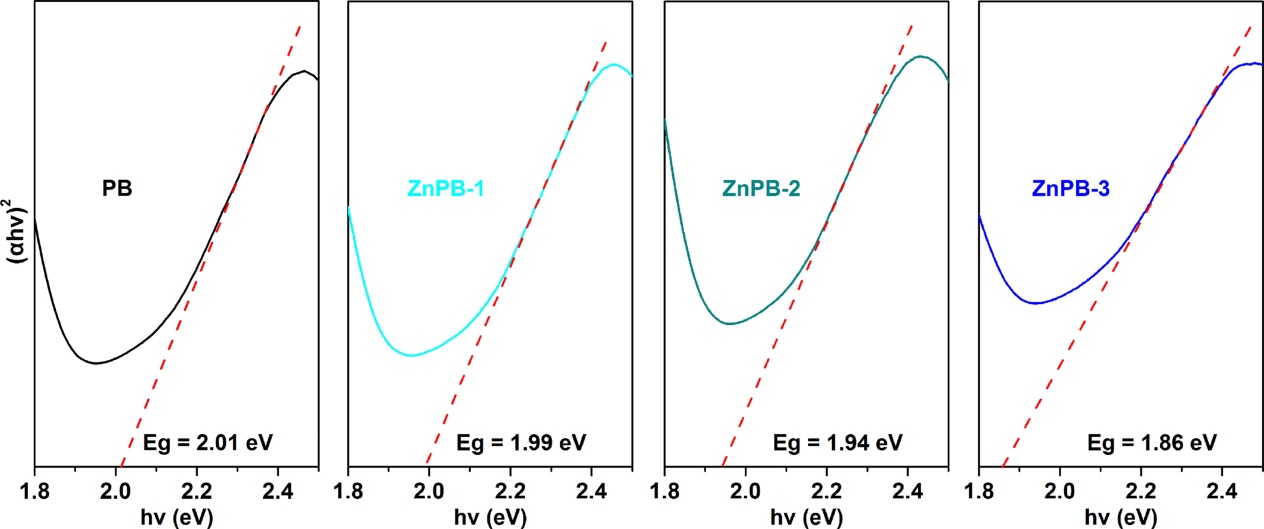


**Supplementary Fig. 6.** Bandgap of PB, ZnPB-1, ZnPB-2, and ZnPB-3 according to the plot of the Kubelka-Munk function versus the bandgap energy from the UV-visible diﬀuse reflectance spectra. Source data are provided as a Source Data file.


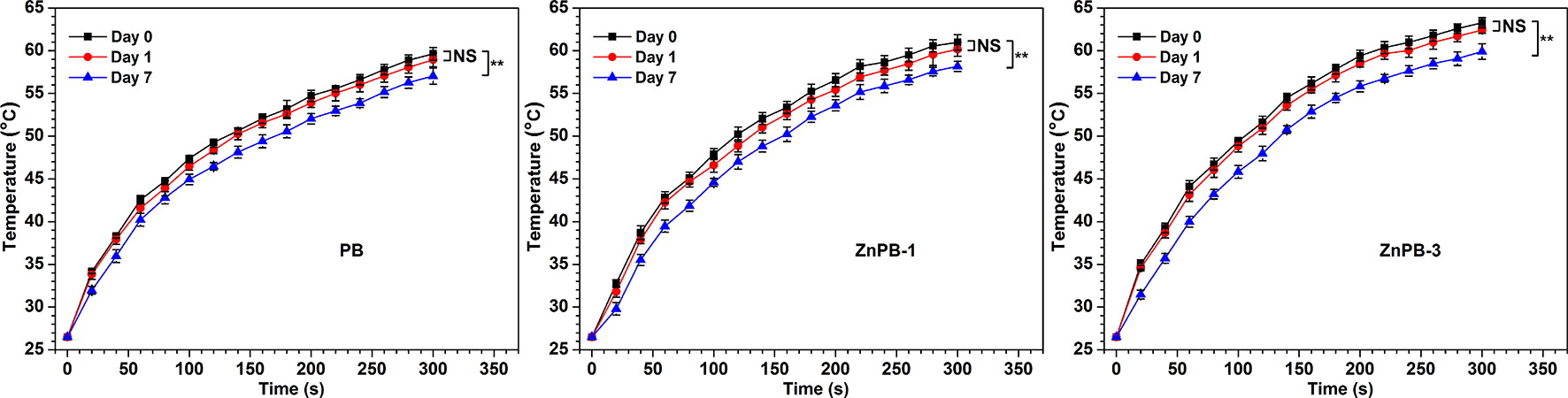


**Supplementary Fig. 7.** Photothermal heating curves of PB, ZnPB-1, and ZnPB-3 solution irradiated with the 808 nm laser (1.2 W cm^-2^) for 5 min are performed for different periods. Error bars indicate means ± standard deviations (n = 3 independent experiments): ***P* < 0.01, (t test). NS, not significant (*P* > 0.05). Source data are provided as a Source Data file.


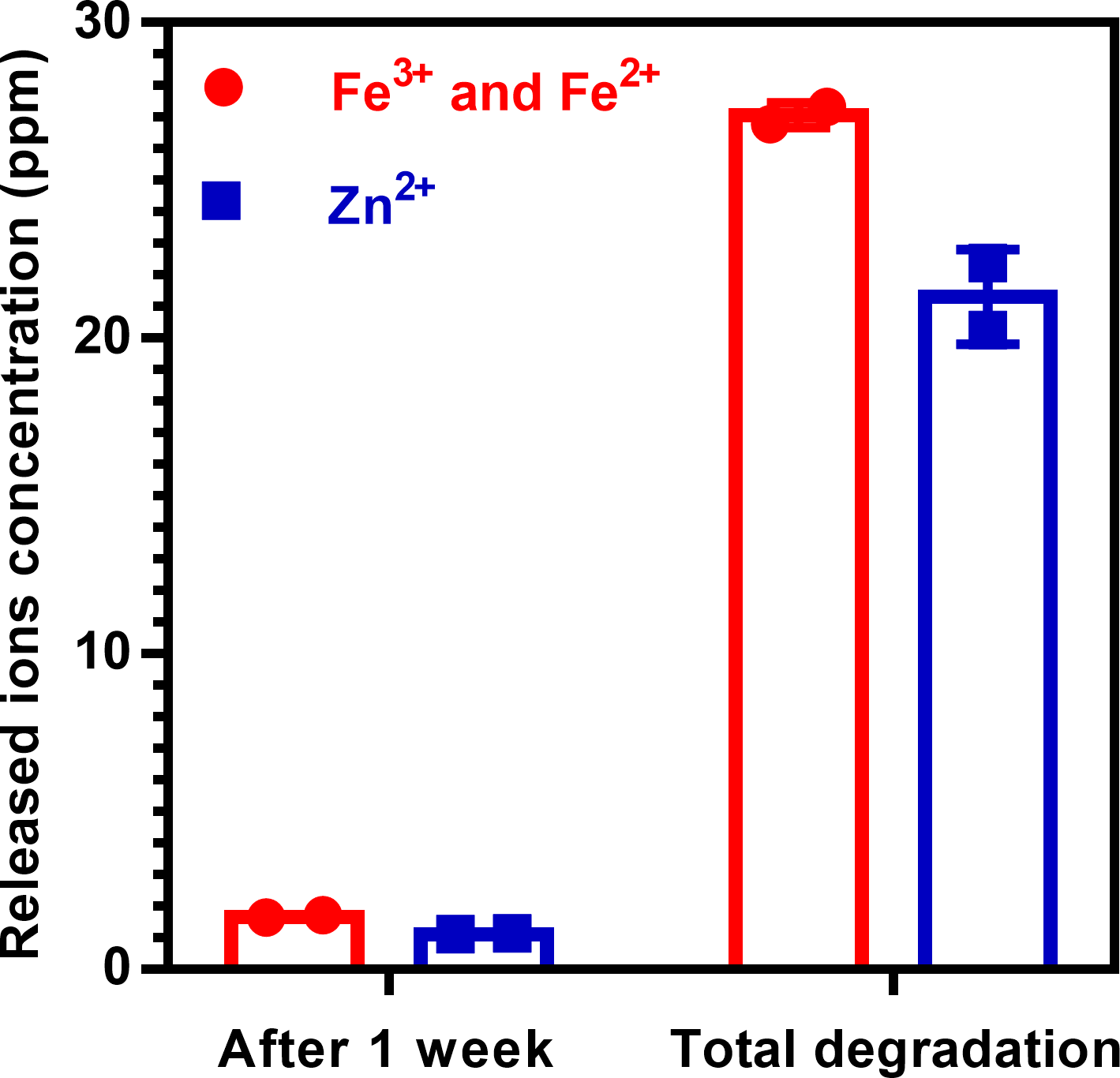


**Supplementary Fig. 8.** Complete degradation of 200 ppm ZnPB-3 solution in sodium hydroxide solution and the cumulative released amounts of Fe^2+^, Fe^3+^, and Zn^2+^ from 200 ppm ZnPB-3 solution in PBS for 1 week. Error bars indicate means ± standard deviations (n = 2 independent samples). Source data are provided as a Source Data file.


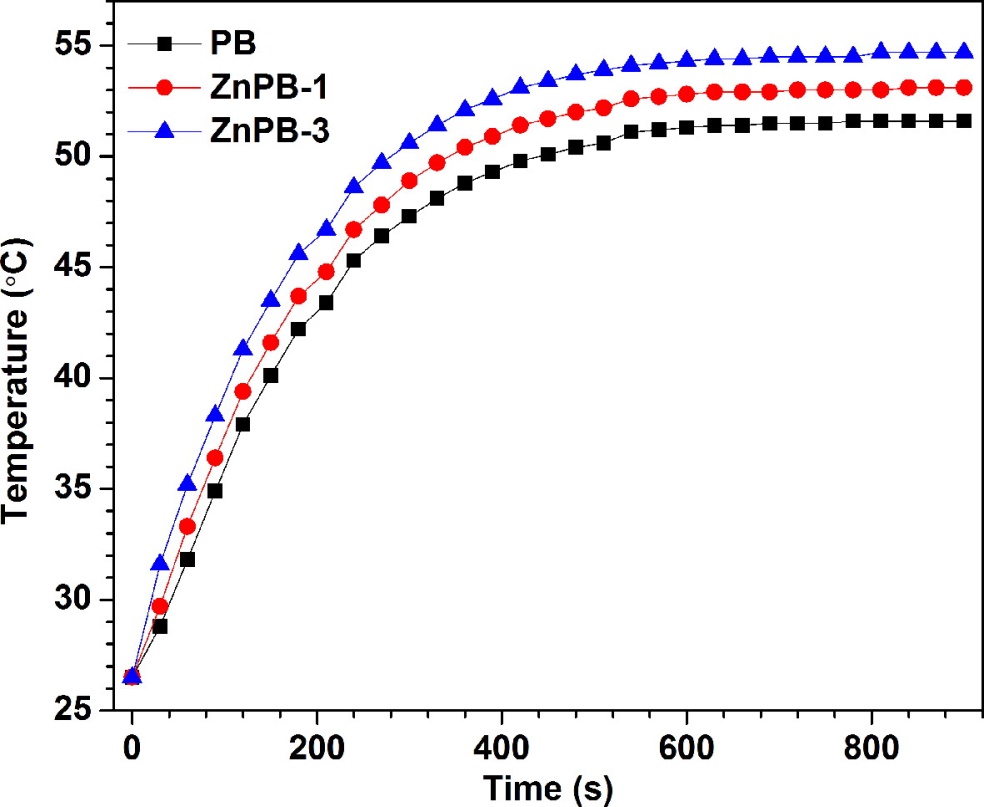


**Supplementary Fig. 9.** Photothermal heating curves of the appropriate photothermal therapy for bacterial infection under 808 nm NIR light irradiation (0.3 W cm^-2^) for 15 min. Source data are provided as a Source Data file.


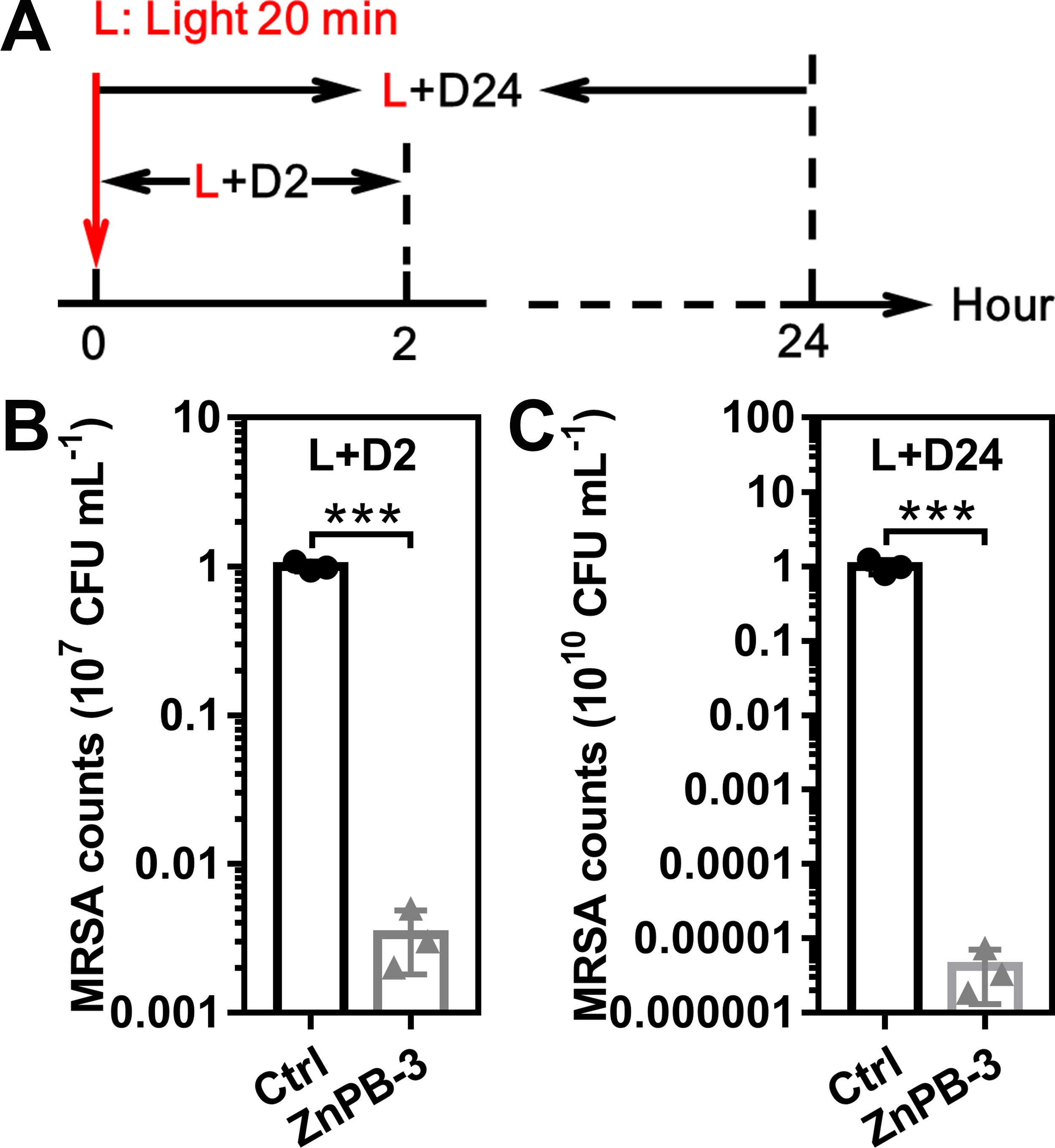


**Supplementary Fig. 10. (A)** Schema of 808 nm light irradiation for 20 min with subsequent culture in the dark for 2 h and 24 h, respectively. Viability of MRSA treated with of ZnPB-3 (200 ppm) under 808 nm NIR light irradiation for 20 min with subsequent culture in dark for **(B)** 2 h and **(C)** 24 h, respectively. Error bars indicate means ± standard deviations (n = 3 biologically independent samples): ****P* < 0.001 (t test). Source data are provided as a Source Data file.

**
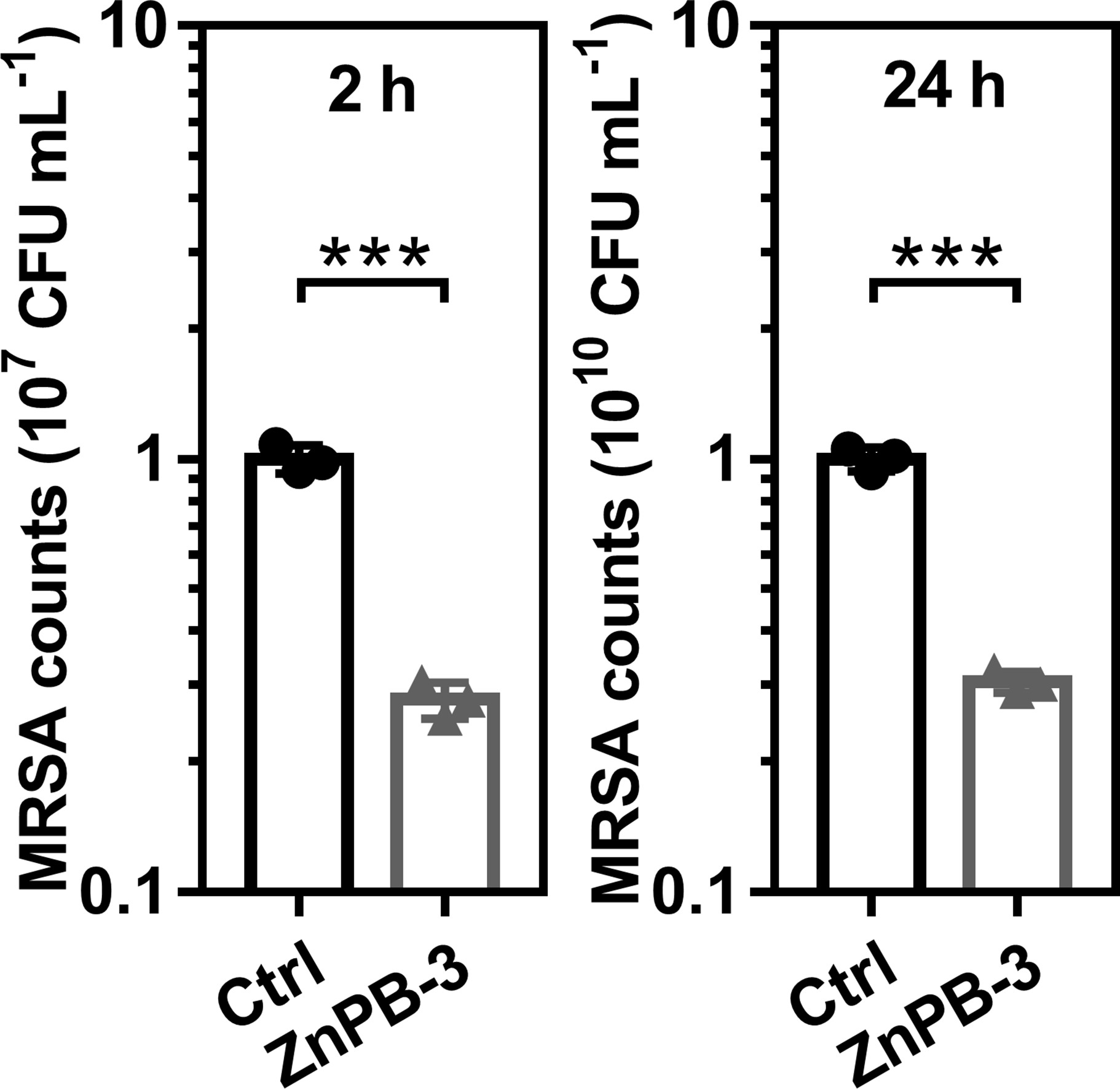
**

**Supplementary Fig. 11.** Viability of MRSA of separated incubation for 2 h and 24 h in dark without the effect of ion release after L treatment with ZnPB-3 (200 ppm). Error bars indicate means ± standard deviations (n = 3 biologically independent samples): ****P* < 0.001 (t test). Source data are provided as a Source Data file.

**
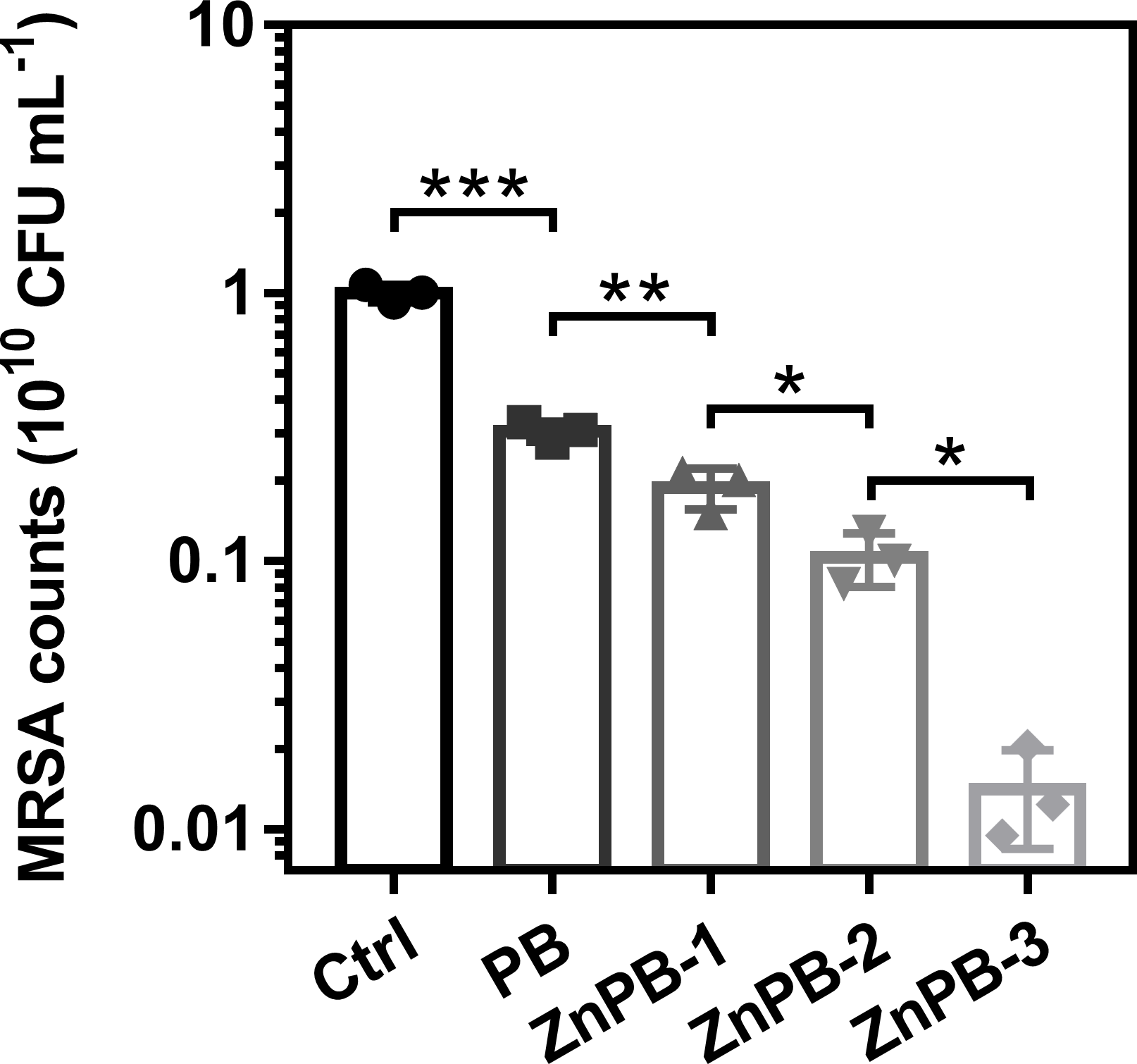
**

**Supplementary Fig. 12.** Viability of MRSA treated with PB, ZnPB-1, ZnPB-2, and ZnPB-3 against MRSA in L+D24 treatment. Error bars indicate means ± standard deviations (n = 3 biologically independent samples): **P* < 0.05, ***P* < 0.01, ****P* < 0.001 (t test). Source data are provided as a Source Data file.

**
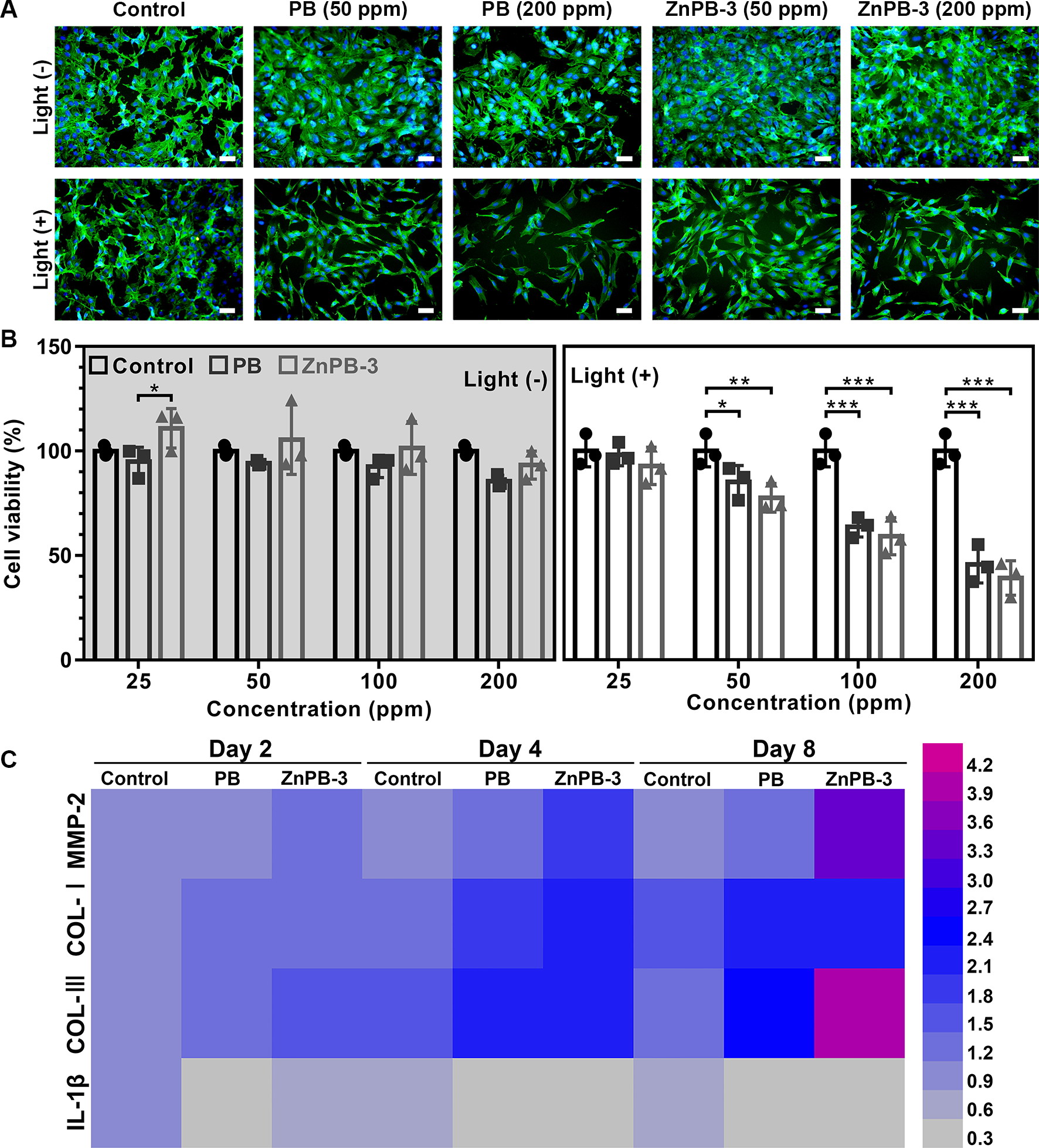
**

**Supplementary Fig. 13.** **(A)** Fluorescent images of NIH3T3 cells after co-cultured with PB and ZnPB-3 (50 and 200 ppm) without or with 808 nm NIR light irradiation and incubation for 24 h at 37 °C; F-actin stained with FITC (green) and nucleus stained with DAPI (blue) (scale bars = 50 μm). **(B)** Cell viability of NIH3T3 cells cultured with PB and ZnPB-3 at various concentrations (25, 50, 100, and 200 ppm) without or with 808 nm NIR light irradiation and incubation for 24 h at 37 ^o^C. **(C)** Fibroblast-related gene expression levels of *MMP-2,* *COL-I*, *COL-III*, and *IL-1β* of NIH-3T3 cells co-cultured with PB (200 ppm) and ZnPB-3 (200 ppm) at 2, 4, and 8 days and evaluated by qRT-PCR using normalization against a *β-actin* reference. Error bars indicate means ± standard deviations (n = 3 biologically independent samples): **P* < 0.05, ***P* < 0.01, ****P* < 0.001 (t test). Source data are provided as a Source Data file.

**
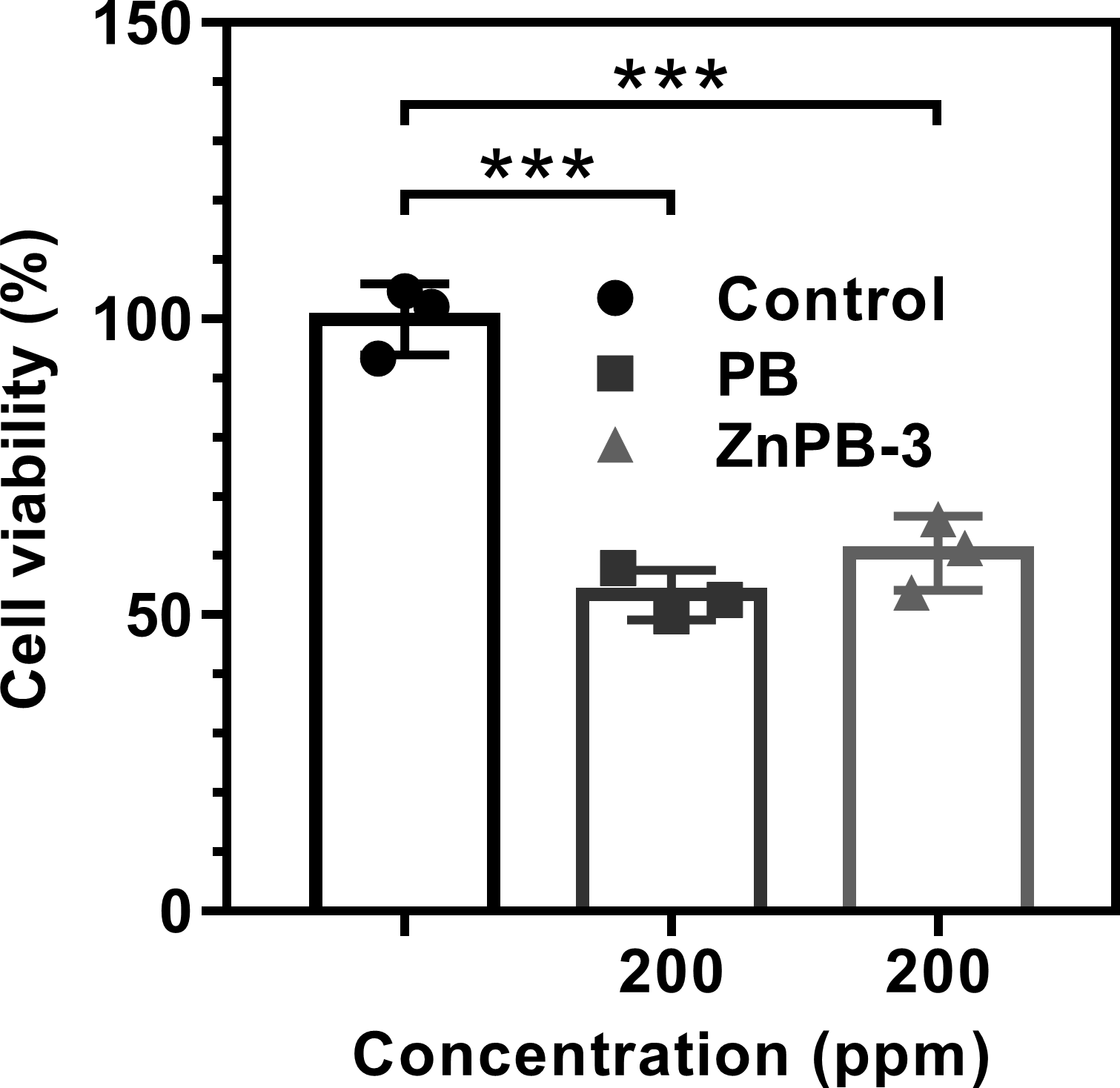
**

**Supplementary Fig. 14.** Cell viabilities of 200 ppm PB group and 200 ppm ZnPB-3 group with 808 nm NIR light irradiation and incubation for day 3. Error bars indicate means ± standard deviations (n = 3 biologically independent samples): ****P* < 0.001 (t test). Source data are provided as a Source Data file.


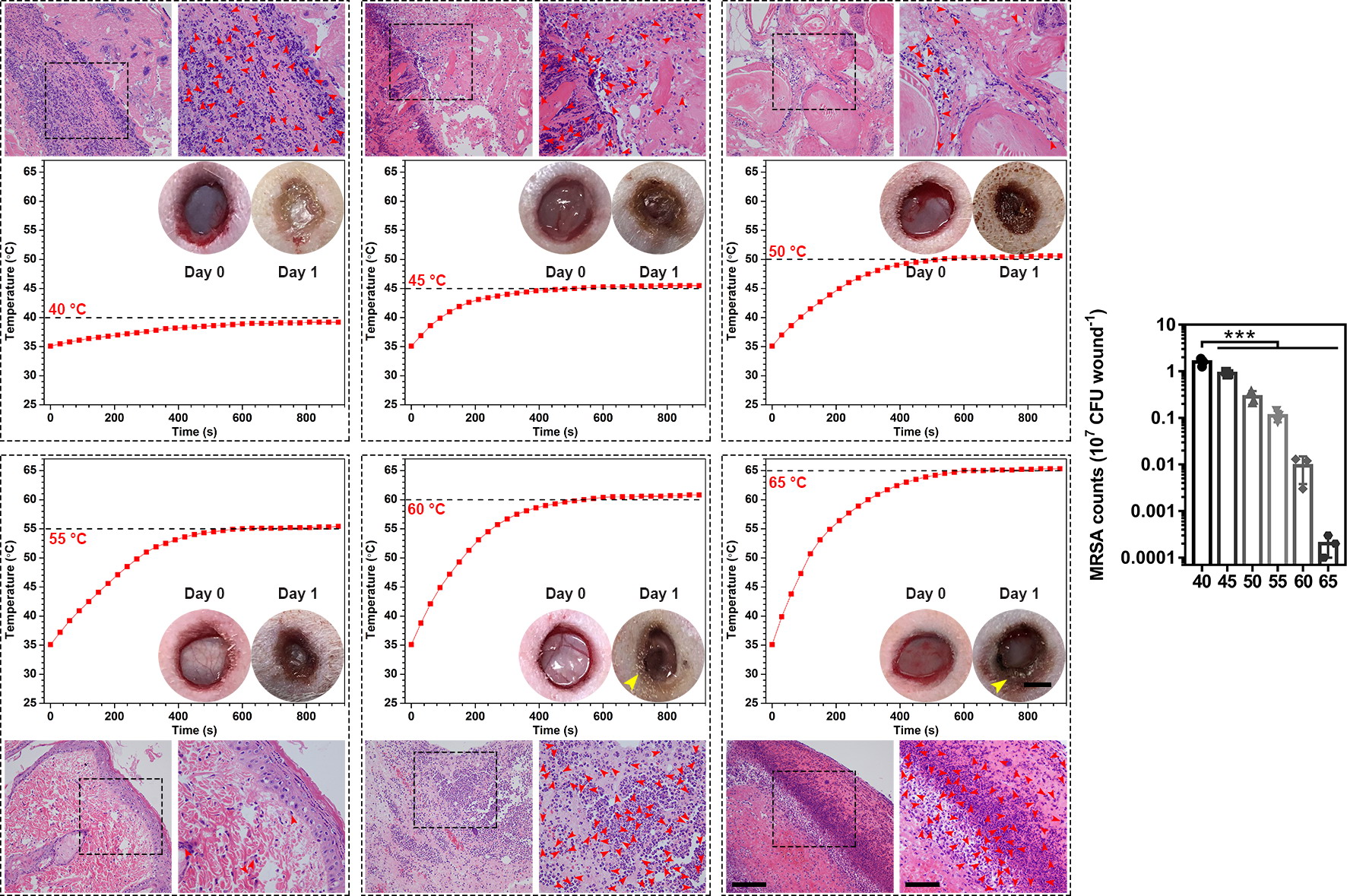


**Supplementary Fig. 15.** Photothermal heating curves of range of temperatures with ZnPB-3 for 15 min, and corresponding photographs (scale bars = 3 mm), H&E staining (scale bars = 100 μm and 50 μm, respectively) of wounds on day 1 and corresponding viability of MRSA. Red arrows mark lobulated neutrophils. Error bars indicate means ± standard deviations (n = 3 biologically independent samples): ****P* < 0.001 (t test). Source data are provided as a Source Data file.


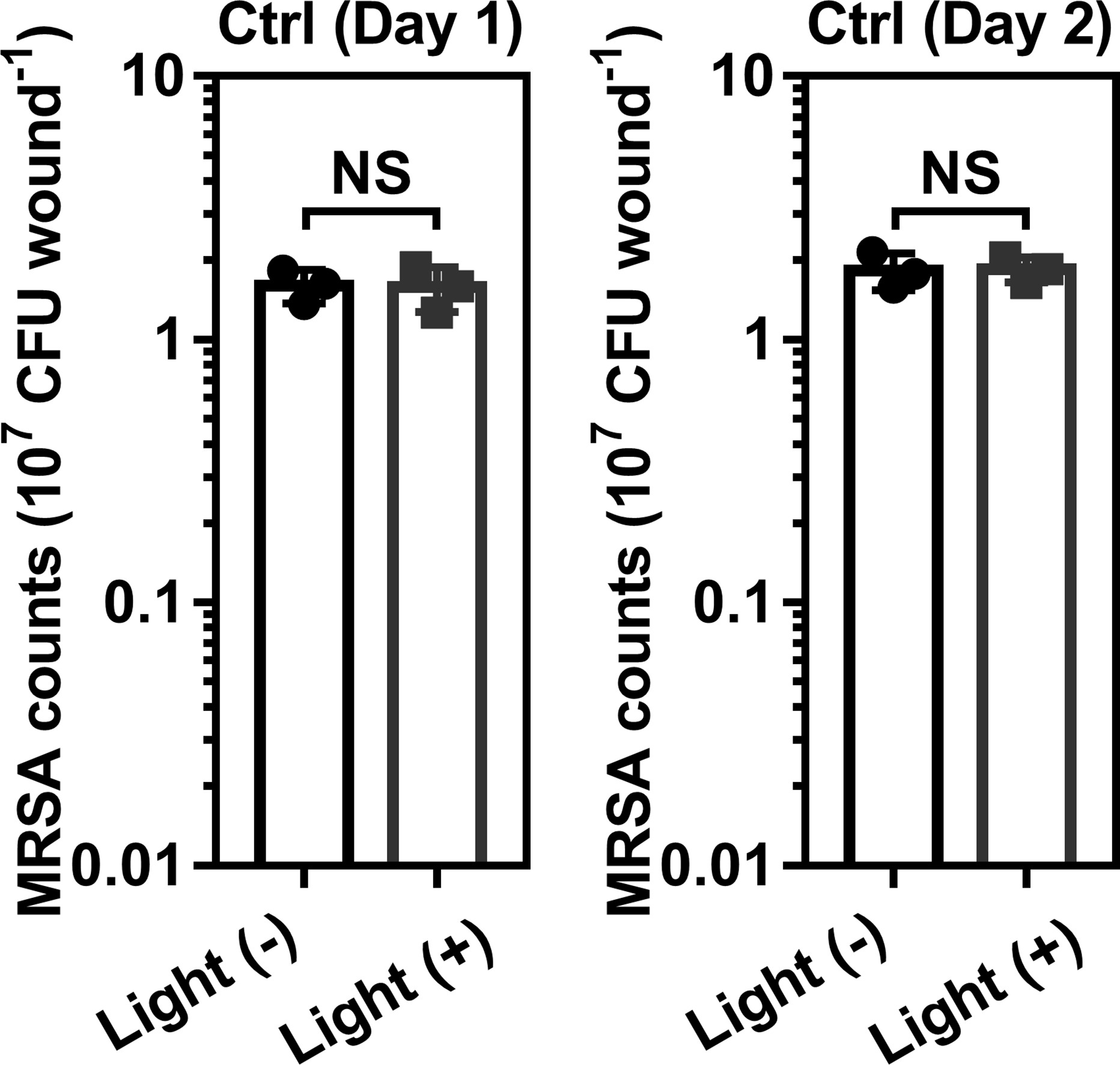


**Supplementary Fig. 16.** Viability of MRSA in control (Ctrl, PBS) groups without and with 808 nm light irradiation for 15 min with subsequent culture in the dark for 1 day and 2 days, respectively. Error bars indicate means ± standard deviations (n = 3 biologically independent samples). NS, not significant (*P* > 0.05). Source data are provided as a Source Data file.


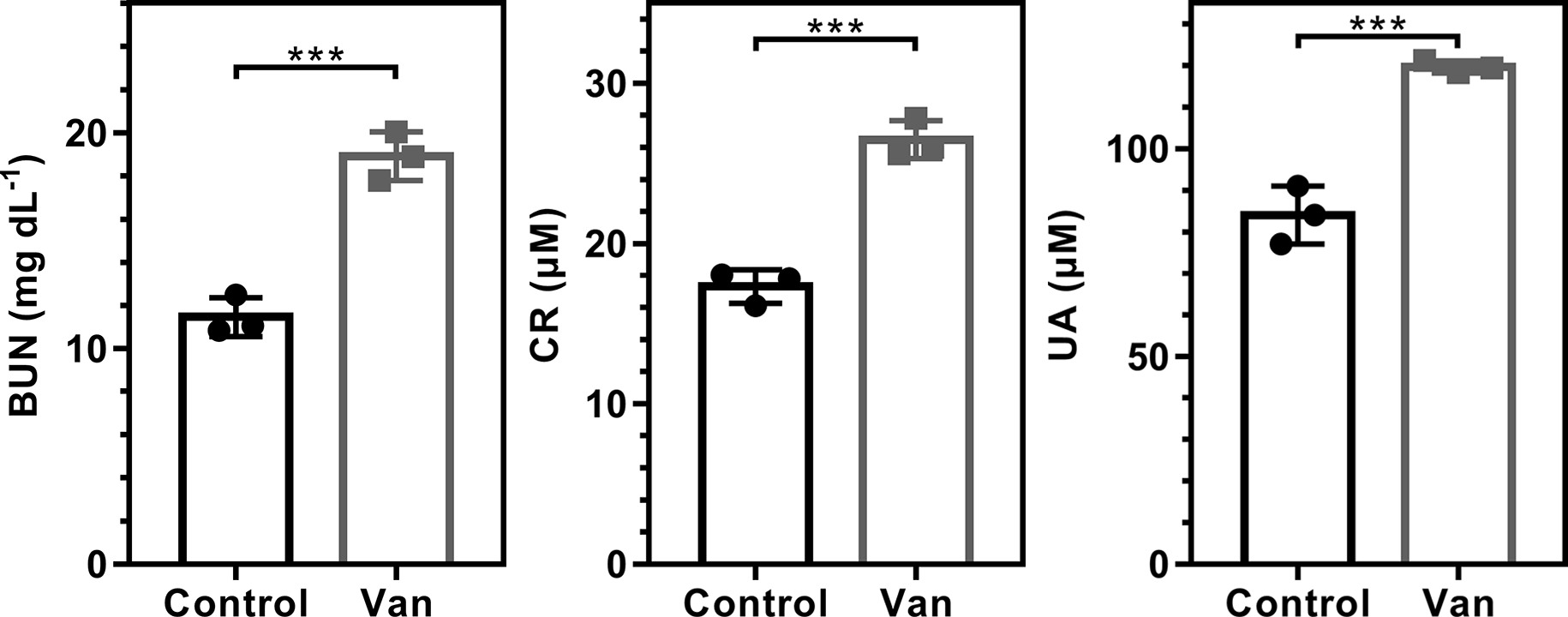


**Supplementary Fig. 17.** Biochemical analyses of renal function (BUN, CR, and UA) of Van in high concentration (160 mg kg^-1^ day^-1^) on day 7. Error bars indicate means ± standard deviations (n = 3 biologically independent samples): ****P* < 0.001 (t test). Source data are provided as a Source Data file.

**
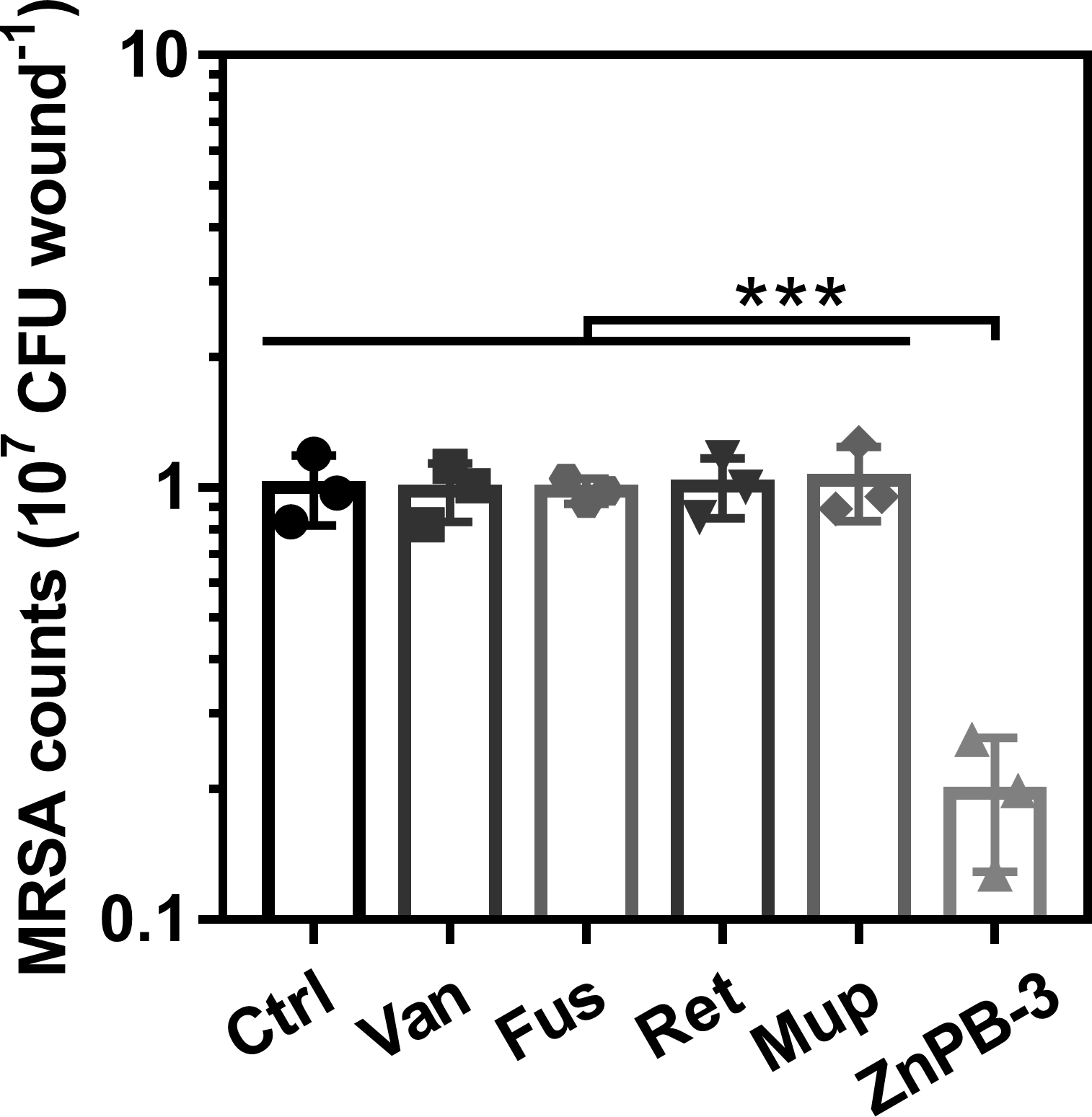
**

**Supplementary Fig. 18.** Viability of MRSA treated with Van (160 mg kg^-1^ day^-1^), Fus, Ret, Mup, and ZnPB-3 using same dosage (0.2 mg kg^-1^ day^-1^) with 808 nm light irradiation for 15 min *in vivo*. Error bars indicate means ± standard deviations (n = 3 biologically independent samples): ****P* < 0.001 (t test). Source data are provided as a Source Data file.


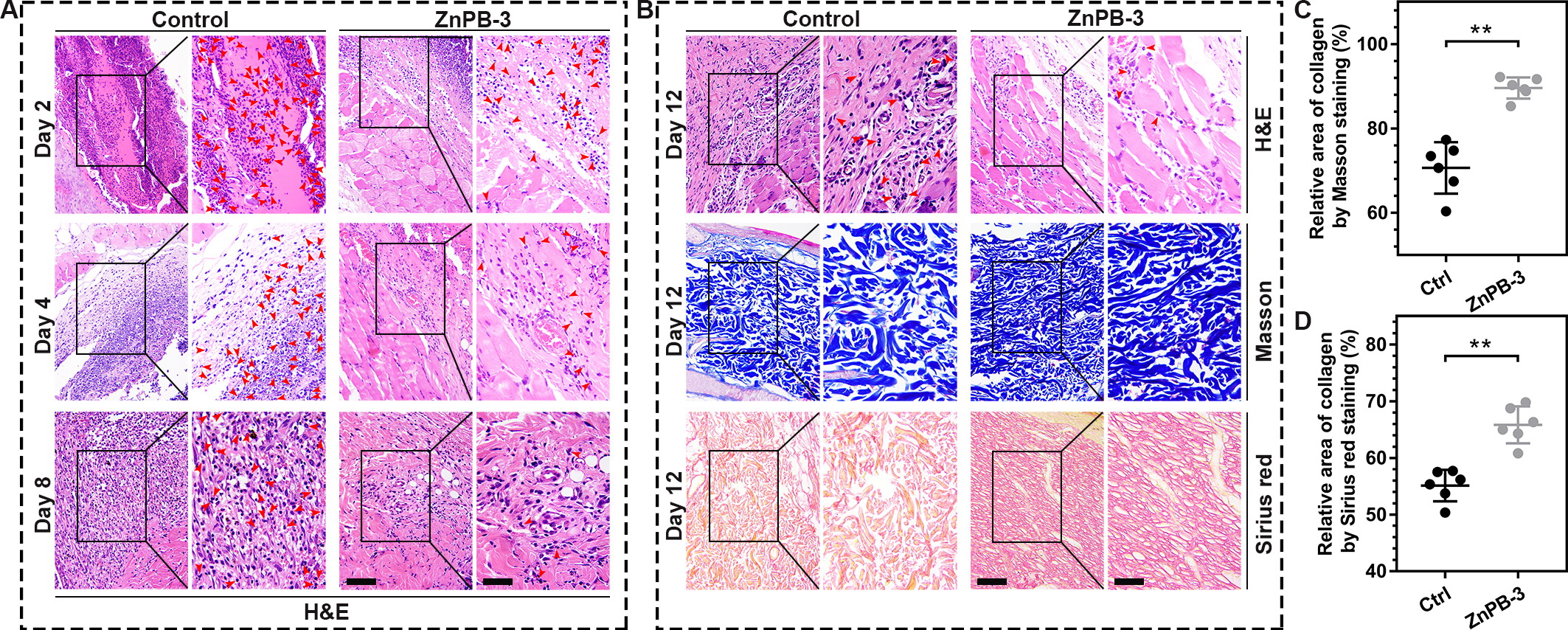


**Supplementary Fig. 19.** **(A)** H&E staining (arrows indicate neutrophils) of control and ZnPB-3 groups at 2, 4, and 8 days at lower and higher magnification (scale bars = 100 μm and 50 μm, respectively); Red arrows mark lobulated neutrophils. **(B)** H&E staining, Masson's trichrome staining and Sirius red staining of control and ZnPB-3 groups at 12 days at lower and higher magnification (scale bars = 100 μm and 50 μm, respectively). Quantitatively relative area of collagen in corresponding **(C)** Masson's trichrome staining and **(D)** Sirius red staining. Error bars indicate means ± standard deviations (n = 6 independent samples): ***P* < 0.01 (t test). Source data are provided as a Source Data file.

**
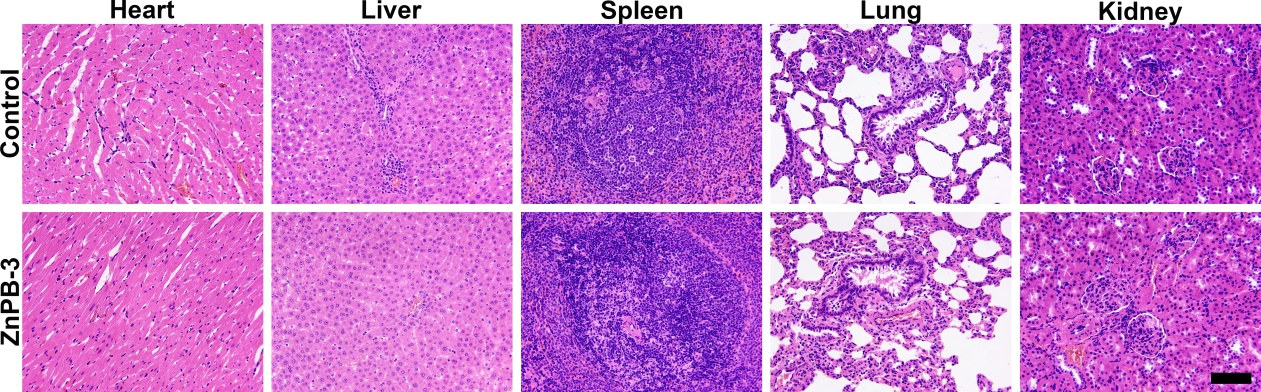
**

**Supplementary Fig. 20.** Histological analyses of major organs (liver, spleen, kidney, heart, and lung) on day 12 through H&E staining (scale bar = 100 μm).


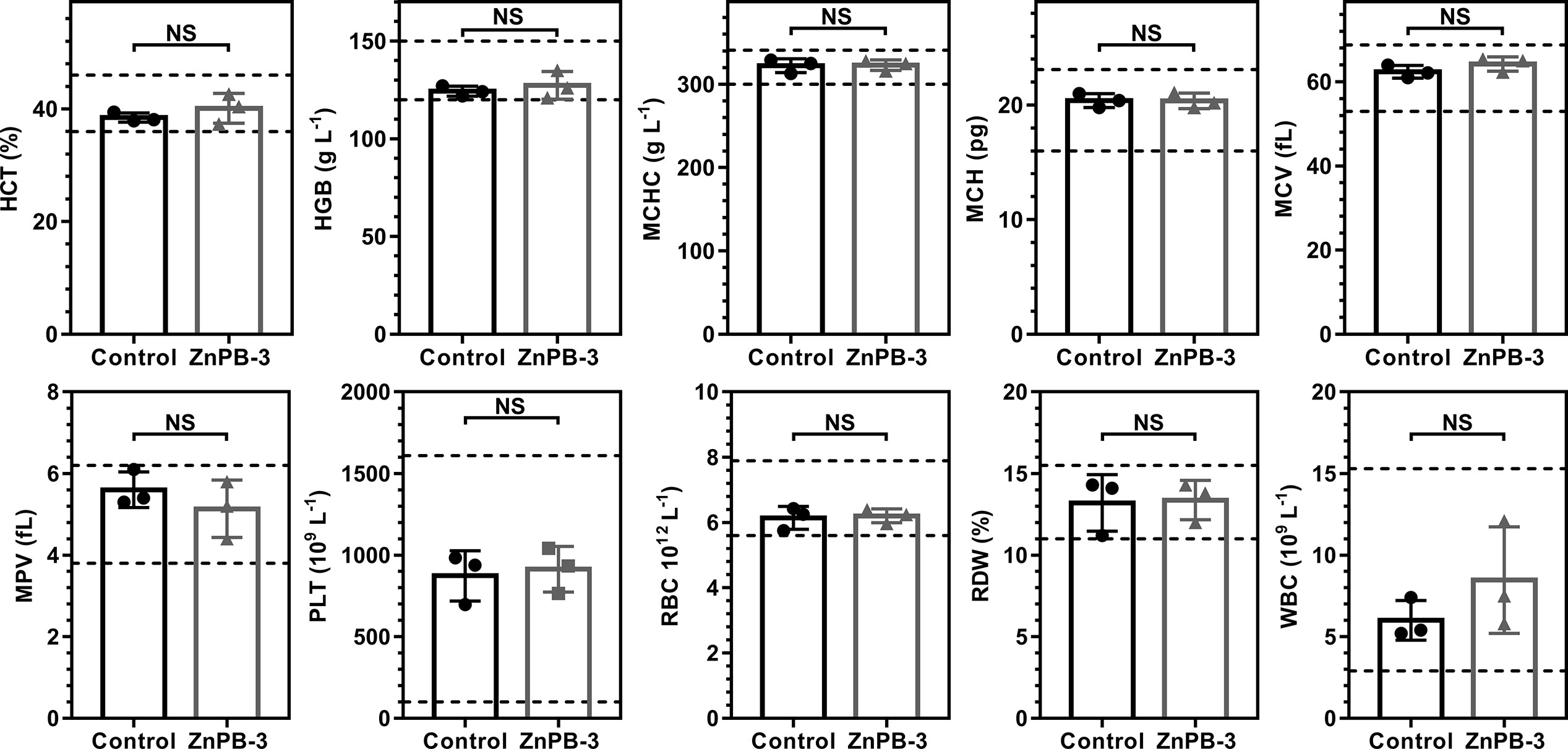


**Supplementary Fig. 21.** Data of standard hematology of control and ZnPB-3 groups on day 12, including HCT, HGB, MCHC, MCH, MCV, MPV, PLT, RBC, RDW, and WBC. Lines of dashes mark corresponding normal scope. Error bars indicate means ± standard deviations (n = 3 biologically independent samples). NS, not significant (*P* > 0.05). Source data are provided as a Source Data file.


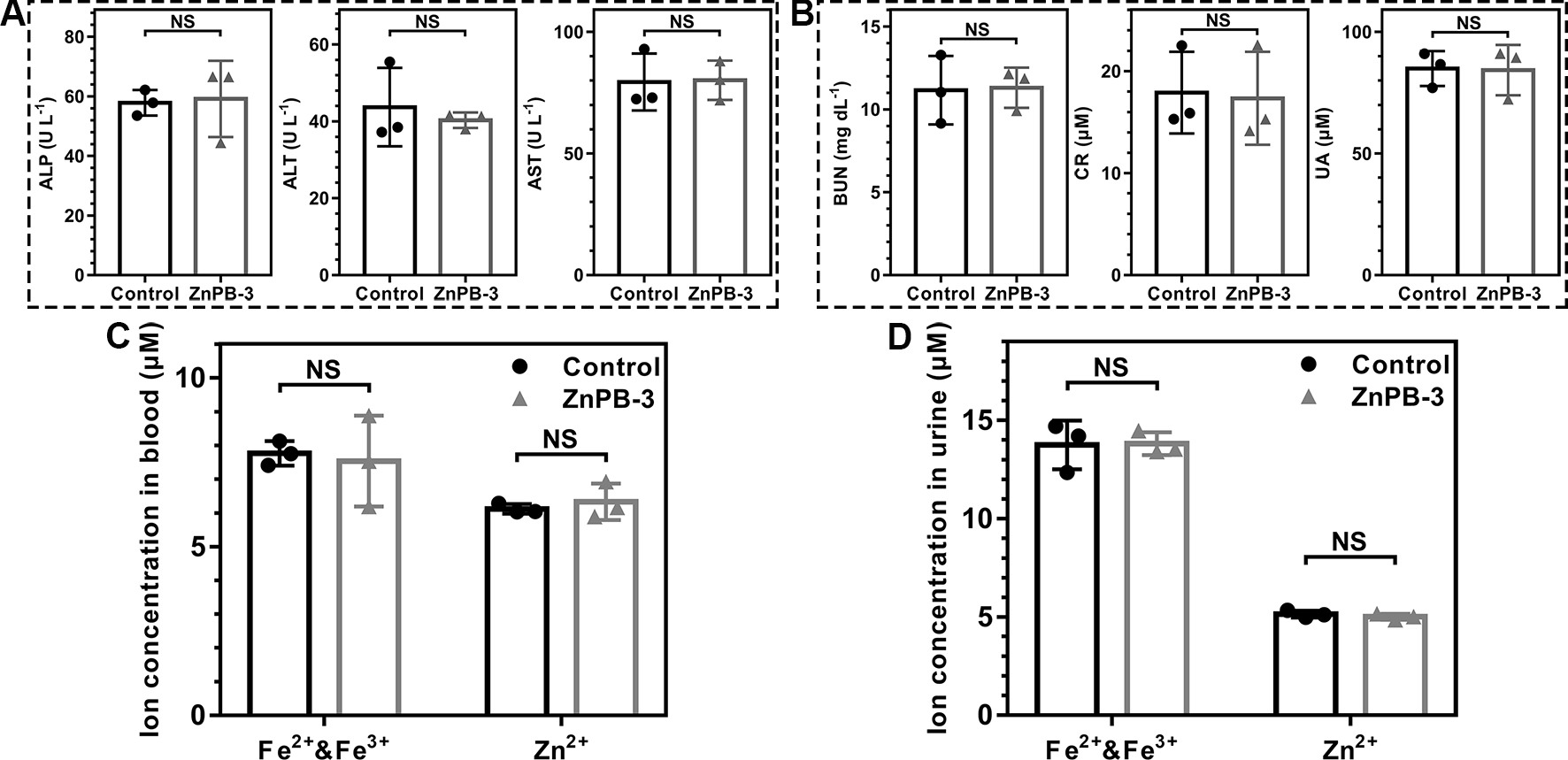


**Supplementary Fig. 22.** Biochemical analyses of **(A)** hepatic function (ALP, ALT, and AST) and **(B)** renal function (BUN, CR, and UA) of control and ZnPB-3 groups on day 12. Concentrations of ions (Fe^2+^, Fe^3+^, and Zn^2+^) **(C)** in blood and **(D)** in urine of control and ZnPB-3 groups on day 12. Error bars indicate means ± standard deviations (n = 3 biologically independent samples). NS, not significant (*P* > 0.05). Source data are provided as a Source Data file.

**Supplementary Tables**

**Supplementary Table 1. The optimized geometrical lattice parameters and its energy of PB, ZnPB-1, ZnPB-2 and ZnPB-3.**

| **Compound** | **a = b = c (Å)** | **α = β = γ** | **Unit-cell volume (Å^3^)** | **Energy (eV)** |
| --- | --- | --- | --- | --- |
| PB | 10.05960 | 90 | 1017.986739 | -410.38462704 |
| ZnPB-1 | 9.998698 | 90 | 999.609235 | -455.56219590 |
| ZnPB-2 | 10.132002 | 90 | 1039.944088 | -443.79413765 |
| ZnPB-3 | 10.2778507 | 90 | 1085.692819 | -432.77556595 |

**Supplementary Table 2. Specific forward and reverse primer sequences of *β-actin*, *IL-1β*, *MMP-2*, *COL-I* and** ***COL-III* genes.**

| Gene | Gene forward primer sequence (5’–3’) | Reverse primer sequence (5’–3’) |
| --- | --- | --- |
| *β-actin* | TGCTATCCAGAAAACCCCTCAA | GCGGGTGGAACTGTGTTACG |
| *IL-1β* | GCTTCAGGCAGGCAGTATC | AGGATGGGCTCTTCTTCAAG |
| *MMP-2* | CTTCCAAGTCTGGAGCGATGT | TACCGTCAAAGGGGTATCCAT |
| *COL-I* | TGCTGATGGACAACCTGG | ACTGTTGCCTTTGGGACC |
| *COL-III* | CTGGATCTCCTGGTGCTAAG | CAGCGTGTCCTTGTGGTC |
