# Supplementary material for: Zinc-doped Prussian blue enhances photothermal clearance of Staphylococcus aureus and promotes tissue repair in infected wounds
Source: Nat Commun. 2019 Oct 3;10:4490. doi: 10.1038/s41467-019-12429-6 (PMC6776522; doi:10.1038/s41467-019-12429-6)
Supplement: Supplementary file 2 — Description of Additional Supplementary Files [file 41467_2019_12429_MOESM2_ESM.docx]

**Description of Additional Supplementary Files**

**File Name: Supplementary Data 1**

**Description:** (1) The optimized parameters of geometric structure of PB ("Occ." is "Occupation"; "Sym." is "Symmetry").

(2) The optimized parameters of geometric structure of ZnPB-1 ("Occ." is "Occupation"; "Sym." is "Symmetry").

(3) The optimized parameters of geometric structure of ZnPB-2 ("Occ." is "Occupation"; "Sym." is "Symmetry").

(4) The optimized parameters of geometric structure of ZnPB-3 ("Occ." is "Occupation"; "Sym." is "Symmetry").
